# Effect of the Long-Term Oral Consumption of Brazil Nut Oil on Adiposity and Glucose Homeostasis in MSG-Induced Obese Male and Female Rats

**DOI:** 10.3390/biomedicines14102284

**Published:** 2026-10-09

**Authors:** Beatriz Machado Daudt, Marianela Andrea Diaz Urrutia, Zoé Maria Neves de Carvalho, Vanessa Marieli Ceglarek, Ellen Carolina Zawoski Gomes, Paulo Cezar de Freitas Mathias, Sabrina Grassiolli

**Affiliations:** 1 Programa de Pós-Graduação em Biociências e Saúde, Centro de Ciências Biológicas e da Saúde, Universidade Estadual do Oeste do Paraná (UNIOESTE), Cascavel 85819-110, PR, Brazilsgrassiolli@gmail.com (S.G.); 2 Programa de Pós-Graduação em Ciências Biológicas, Departamento de Fisiologia, Universidade Federal do Rio Grande do Sul (UFRGS), Porto Alegre 90050-170, RS, Brazil; 3 DOHaD Experimental Laboratory, PhD Molecular and Cell Biology Program, Maringá State University, Maringá 87020-900, PR, Brazil; 4 Multicentre Health Institut, Anízio Teixeira Campus, Federal University of Bahia, Vitória da Conquista 45029-094, BA, Brazil

**Keywords:** adiposity, metabolism, Wistar rats, sex, monosodium glutamate, Brazil nuts

## Abstract

**Background/Objectives**: Brazil nut (*Bertholletia excelsa* (Be)) consumption, in the form of oil or seed, has been associated with health benefits in both the lean and obese conditions; however, the influence of sex on these effects is unknown. We analyzed the effects of oral Be-oil supplementation on adiposity and glucose homeostasis in hypothalamic obese and lean male and female rodents. **Methods**: Male and female Wistar rats were submitted to hypothalamic lesions by monosodium glutamate (MSG; 4 g/kg). An equimolar saline solution was administered to lean, or control (CON), rats. The MSG (obese) and CON (lean) males and females were subdivided into Be-oil-supplemented (1 mL/kg; 3 times/week/8 weeks) and non-supplemented (Ns; saline) groups. Food consumption, feed efficiency ratio, body weight, and white and brown adipose tissue (WAT/BAT) content were recorded. Fasting values of glucose, triglycerides, total cholesterol, and insulin resistance (IR) were assessed. **Results**: MSG-obese rats presented increased adiposity, IR, and metabolic dysfunction in both sexes without altering food intake. Independently of sex, long-term oral supplementation with Be-oil did not prevent obesity development and led to reduced feed efficiency. Sex-dependent effects of Be-oil supplementation were noted, with MSG_Be_ males showing slight improvements in glucose control and insulin responsiveness, despite worsened triglycerides and a reduction in islet number in the pancreas. In contrast, although MSG_Be_ females presented reduced visceral WAT hypertrophy, IR worsened without alterations in insulin secretion, islet histology, or oral glucose tolerance. **Conclusions**: In both sexes, oral Be-oil supplementation did not prevent obesity development in MSG-hypothalamic obese rats; however, it exerted sex-dependent metabolic effects, with more positive effects on glucose homeostasis in MSG-obese males.

## 1. Introduction

Obesity is a chronic and complex disease involving alterations in neuronal, hormonal, and metabolic pathways, leading to excessive adiposity, insulin resistance (IR), hyperglycemia, dyslipidemia, and hypertension, as commonly observed in metabolic syn-drome (MS) [1,2]. The prevalence of overweight and obesity continues to rise worldwide, including in Brazil. Among Brazilian adults, obesity increased from 11.8% in 2006 to 20.3% in 2019, and projections for 2030 estimate that 68.1% of the adult population will be overweight and 29.6% will be obese [3]. These data indicate that conventional strategies to control body weight (BW) and prevent fat mass accumulation and related diseases, such as diabetes, remain insufficient [4,5,6]. In this context, natural products rich in bioactive compounds have emerged as alternative strategies to manage obesity and its deleterious health effects [6].

The Brazilian Amazon rainforest harbors a large diversity of plants with medicinal properties, such as *Bertholletia excelsa* (Be), commonly known as the Brazil nut, which has demonstrated positive effects on glucose and lipid homeostasis [7,8,9]. The Brazil nut is a nutrient-rich food containing lipids such as monounsaturated (MUFA) and polyunsaturated (PUFA) fatty acids [10]. Moreover, important micronutrients such as selenium (Se), copper, magnesium, zinc, and vitamins E and C are also present in Brazil nuts [10,11]. As such, due to its composition, regular Be consumption, in the form of oil or seed, may exert beneficial effects on obesity and MS [8,12,13]. A systematic review demonstrated that, both in men and women, the consumption of nuts, including Brazil nuts, may modulate satiety signaling, favoring appetite regulation and glucose homeostasis, reducing cardiovascular risk and diabetes development [8]. Moreover, the nutritional composition of Brazil nuts suggests a potential role in reducing white adipose tissue (WAT) inflammation and increasing energy expenditure, although direct evidence supporting these effects remains limited [12,13].

Beneficial effects of Brazil nut oil or seed consumption have also been observed in several rodent models; however, these effects are displayed preferentially in male rats [14,15,16]. In this regard, recently published data showed that a diet enriched with 5% Brazil nuts for 8 weeks was effective at reducing oxidative stress and protecting cardiovascular function in nephrectomized male Wistar rats [17]. The consumption of Brazil nuts by healthy male rats improved bone parameters, high-density lipoprotein cholesterol (HDL-c) levels, lipid peroxidation, and adiposity, responses that were all dependent on the percentage of Brazil nuts in the diet [14]. Anti-adiposity effects, lower serum glucose levels, and improved vascular function were also observed in hypertensive male Wistar rats supplemented with Be [15]. Positive effects of Brazil nut supplementation were also observed in diabetic male rats, including improvements in reproductive parameters and antioxidant capacity [16].

However, the absence of, or negative, impact of Be consumption in both sexes has also been reported [18,19,20]. For example, acute effects of cashew and Brazil nut consumption on appetite not were observed in either women or men [18]. In obese women, Brazil nut consumption increased gene expression of several pro-inflammatory biomarkers, including interleukin-6 (IL6) and tumor necrosis factor-α (TNF-α) [21]. Moreover, Be-oil supplementation augments plasmatic triglyceride levels in women with MS [10]. These findings indicate the need to expand experimental studies with Be supplementation, especially using diverse obesity models and assessing sex influences.

Food intake and energy expenditure are regulated by hormonal and neuronal peripheral pathways coordinated by hypothalamic neurons [22]. In rodents, neonatal administration of high doses of monosodium glutamate (MSG) induce hypothalamic lesions, particularly in the arcuate nucleus (ARC) [23]. MSG-treated rats develop autonomic and hormonal dysfunctions—marked adiposity with increased WAT, IR, glucose intolerance, dyslipidemia, and cardiovascular impairments—without hyperphagia [23,24,25]. However, despite excessive WAT mass and in contrast to other obese models (e.g., high-fat diet or genetic), MSG-induced obesity is not associated with augmented body weight [26]. This profile is attributed to reduced muscle and bone mass as a consequence of diminished circulating growth hormone (GH) levels after ARC lesions [26].

Therefore, MSG-induced obesity may induce may similar features to those observed in hypothalamic obesity in humans, including rapid weight gain despite limited food intake, central insulin and leptin resistance, reduced sympathetic activity, low energy expenditure, and increased WAT accumulation [27]. Moreover, the reduced muscle mass observed is also similar to that seen in the sarcopenia model, a condition commonly found in obese humans and related to several metabolic dysfunctions [28]. In both male and female rodents, MSG treatment also causes gonadal atrophy and hormonal dysfunctions, making it important to evaluate sex influences in anti-adiposity treatments [29].

In humans, sex strongly influences adiposity by modulating adipogenesis and metabolic profiles [30]. Sex-related differences are also evident in MSG-induced obesity in rodents [31,32] and would influence responses to Be-oil supplementation, which is an unknown effect. Moreover, it has been previously demonstrated that the manner in which nuts are consumed may interfere with their effects [33]. Herein, Be-oil form was adopted to avoid the well-recognized influence of the masticatory and hormonal incretins in metabolism, which are more difficult to evaluate in rodents. In addition, this form guarantees an adequate dose, avoiding inhibitory fiber effects on lipids absorption, as previously demonstrated for Brazil nut seed consumption [34]. Thus, our hypothesis was that oral Be-oil supplementation would induce sex-dependent metabolic and adiposity effects, with more pronounced benefits in obese animals.

Therefore, the aim of this study was to assess the effects of oral Be-oil supplementation on adiposity and metabolic parameters in MSG-induced obese and non-obese rodents, considering sex influences.

## 2. Materials and Methods

### 2.1. Animals and Ethics Statements

Rats were housed in polypropylene cages properly lined with wood shavings and maintained under controlled conditions, on a 12 h light/dark cycle, with constant temperature (23 ± 2 °C), receiving food and water ad libitum during lactation and throughout the growth period, according to the Brazilian National Council for the Control of Animal Experiments (in Portuguese, Conselho Nacional de Controle de Experimentação Animal—CONCEA) guidelines and based on international standards for animal care and maintenance [35]. The experimental protocols were approved by the Animal Ethics Committee (CEUA 13-20) of the Universidade Estadual do Oeste do Paraná (UNIOESTE). At birth, offspring size was adjusted to 6–8 pups per dam (*n* = 3–4 male rats and *n* = 3–4 female rats) to guarantee an adequate nutritional supply during lactation.

### 2.2. Hypothalamic Obesity Induction

Hypothalamic lesions were induced in males (*n* = 30) and females (*n* = 40) through subcutaneous injections of commercial MSG (4 g/kg BW; Ajinomoto Co., São Paulo, Brazil), which was administered for five consecutive days, from the second to the sixth postnatal day (PND), according to the Olney’s adapted protocol [36]. At the same time, non-obese or control (CON) groups (*n* = 30 male rats and *n* = 40 female rats) received subcutaneous injections of equimolar NaCl solution (1.25 g/kg BW). MSG and CON rats of both sexes were weaned on the 21st day of age and were allocated following a randomized sequence according to sex, treatment, and supplementation, then regrouped into 3–4 rats per cage, according to these experimental characteristics.

### 2.3. Be-Oil Supplementation

At PND 30, half of the animals CON (*n* = 15–20 rats/sex) and MSG (*n* = 15–20 rats/sex) groups were submitted to Be-oil supplementation offered orally via the gavage method, at a dose of 1 mL/kg BW, which was administered 3 times/week for 8 weeks. Similar doses of Be-oil supplementation were previously used in fish [37], pregnant rats [38] and humans [10], with no toxic effects registered. The Be-oil offered to supplemented groups was extracted from the seeds of Be (AmazonOil Company, Ananindeua, Pará, Brazil). Certifications of analysis and fatty acid composition are available on the website: https://www.amazonoil.com.br/pt/castanha-do-para/ (accessed on 7 July 2026) and are summarized in the Supplementary Data (Table S1). Table S1 also shows the estimated human equivalent dose (HED), as proposed by Nair and Jacob [39] and shows the Se concentration in Be-oil, according the calculation of Zhanglin et al. [40]. Non-supplemented (Ns) rats from the CON (*n* = 15–20 rats/sex) and MSG groups (*n* = 15–20 rats/sex) received, by the same route and with the same frequency, an equivalent volume of saline solution (0.9% NaCl). Other oils were not used as a negative control to avoid any effects on metabolism or intestinal motility and absorption [41]. Thus, considering MSG obesity induction, Be-oil supplementation, and sex, eight experimental groups were formed, according to the experimental design shown in Figure 1.

### 2.4. Food Intake, Feed Efficiency Ratio and Body Weight Gain

From PND 30 to 90, all experimental groups received standard chow (BIOBASE, Águas Frias, SC, Brazil) and tap water ad libitum. During this period, each animal was individually identified by tail marking, and body weight (BW) and food consumption were recorded once per week. Food intake was expressed as g/g of BW. To this end, the excess food was subtracted from the total amount of food offered, with this value being divided by the total number of animals per cage and subsequently by the individual BW of the rats. Thus, individual BW and food intake records were used to calculate the respective area under the curve (AUC). Moreover, the feed efficiency ratio (FER) was calculated. For this, BW gain delta (∆) was obtained by subtracting the final BW (at 90 days of age) from the initial BW (at 30 days of age), and this value was divided by the total sum of food intake (FI, ∑), as shown by the formula below [42]: FER: ∆ BW (g)/∑ FI (g).

### 2.5. Oral Glucose Tolerance Test

At PND 88, some animals were randomly separated (4–5 rats per group) and submitted to an oral glucose tolerance test (oGTT). For this, the animals were fasted for 12 h and were placed in individual cages. Next, a blood sample was collected from the tail vein, and fasting glycemia was evaluated using a glucometer (AccuChek Active, Roche Diabetes Care, Jaguaré, Brazil). Subsequently, a glucose overload (2 g/kg BW) was administered orally, and glucose levels were evaluated at 15, 30, 45, 60, and 120 min after the glucose load. The glycemic levels obtained during the oGTT were used to calculate area under the curve (AUC) data. Additionally, we evaluated the pattern of the glucose curve during the oGTT by obtaining the glucose time peak (min) and glucose peak value (mg/dL), according to the protocols of Kramer et al. [43] and Siqueira et al. [44].

### 2.6. Euthanasia, Blood and Tissue Collection

At PND 90, and after 12 h of fasting, BW (g) and naso-anal (NAL; cm) length was measured and Lee index was subsequently calculated using the formula ∛ BW(g)/NAL (cm), as proposed by Bernardis and Patterson [45]. The rats were then euthanized by decapitation after brief CO_2_ desensitization. Trunk blood was immediately collected into heparinized tubes, centrifuged (3000× *g*; 15 min), and plasma was separated for biochemical analysis, as described below. Subsequently, the abdominal cavity was opened, and the pancreas, brown adipose tissue from the interscapular (iBAT) region and perigonadal (P) and inguinal (I) WAT depots were excised, weighed, and stored in ALFAC solution (85%: 70% ethanol, 10%: 37–40% formaldehyde, and 5% glacial acetic acid) for 24 h. Next, the tissues were transferred to 70% ethanol for subsequent histological analysis, as described below. Tissue weights were normalized to individual BW and expressed as g/100 g BW.

### 2.7. Islets Isolation and Glucose-Induced Insulin Secretion

After abdominal laparotomy, some rats (*n* = 4–6/group) were used for islet isolation, according to a protocol previously established by Lacy and Kostianovsky [46], with modifications by Grassiolli et al. [47]. Briefly, immediately after abdominal laparotomy, the pancreas was perfused with Hank’s buffer solution (HBS: 2.8 mM glucose; pH: 7.4; and 0.1% BSA) containing collagenase type V (Sigma-Aldrich, St. Louis, MO, USA; 1 mg/mL). Subsequently, the organ was removed and transferred to a Falcon tube containing 10 mL of HBS in a water bath at 37 °C for approximately 15 min for collagenase digestion. Afterwards, the digested tissue was submitted to several washing steps with cold HBS, followed by centrifugation to obtain a pool of islets. Thus, islets were macroscopically examined with the aid of magnifying glass and manually collected. Four islets were transferred to wells in a culture plate containing 1 mL of Krebs–Ringer buffer (KRB) containing 5.6 mM glucose, 0.1% BSA, and pH: 7.4, under a gas condition (CO_2_, 5%) at 37 °C for 60 min during the pre-incubation phase. Pre-incubation secretion was discarded, and glucose-induced insulin secretion (GIIS) studies were immediately started. For this, islets received 1 mL of KRB (0.1% BSA and pH: 7.4) with glucose under non-stimulatory conditions (5.6 mM), under a gassed condition (CO_2_, 5%) at 37 °C for 60 min. Subsequently, the supernatant was collected and stored at −20 °C. Aliquots of supernatant were used for insulin measurement via radioimmunoassay (RIA) technique using iodine (^125^I), according to the principles originally proposed by Yalow & Solomon, [48]. Briefly, the assay was based upon the competition between hot 125I-insulin (Revvity, Waltham, MA, USA) and plasma rat insulin binding to the limited quantity of antibodies specific for the peptides in each reaction mixture. After 48 h of incubation period, I^125^ was measured using the Wizard Gamma Counter (Perkim Elmer, Norwalk, CT, USA). A standard curve was constructed by plotting the percentage of antibody-bound radiolabeled antigen against known concentrations of a standardized unlabeled antigen, and the concentrations of insulin in samples were extrapolated from that curve [48].

### 2.8. Biochemical Plasma Parameters

The plasma obtained was used to measure blood glucose, triglycerides, and total cholesterol, using colorimetric enzymatic kits (Lab Test, Lagoa Santa, Brazil; GPO-Trinder, Laborclin, Pinhais, Brazil, respectively), the measurements were performed with a spectrophotometer at a wavelength of 490–520 nm, as indicated by the manufacturer’s instructions. The triglyceride–glucose (TyG) index was calculated from the natural logarithm (Ln) of fasting plasma glucose (mg/dL) values multiplied by fasting triglyceride (mg/dL) values divided by two, as indicated by the formula of Simental-Mendía et al. [49]: {Ln (fasting triglycerides mg/dL × fasting glucose mg/dL)}/2. The TyG index is a well-established marker of IR in humans and obese rodents. Using fasting values of glucose and insulin (measured by RIA technique), we performed the Homeostasis Model Assessment of Insulin Resistance (HOMA-IR), which measures insulin resistance and is calculated by multiplying fasting blood glucose (mmol/L) by fasting insulin (µIU/mL) and dividing by 22.5. HOMA-beta (β) was also calculated to assess pancreatic beta cell function, using the following formula: (20 × fasting insulin µIU/mL)/(fasting blood glucose mmol/L − 3.5); both indices were calculated according to Matthews et al. [50].

### 2.9. Histological Analysis

Samples (*n* = 5–6 animals/group) of pancreas, WAT (I and P), and iBAT were submitted to histological procedures and image analysis. For this, the samples were dehydrated in increasing alcohol concentrations, diaphanized in xylol, and embedded in paraffin (ACS Científica, São Paulo, Brazil). The samples were then sectioned with a microtome (5–7 µm; OLYMPUS CUT4055, Olympus, Tokyo, Japan), and three slides per animal were prepared with 2–3 sections/slide. The sections were stained with hematoxylin and eosin (H&E), and photomicrographs were recorded with a DP71 controller (Olympus, Tokyo, Japan). Quadrants (40×) were pre-selected in WAT (P and I) and in iBAT, and the number of nuclei and the area of adipocytes were measured. For the pancreas, photomicrographs were recorded at 20×, and the area and number of pancreatic islets were measured. All image areas were evaluated using the polygonal measurement tool in Image-Pro Plus software (v4.5) and calibrated according to the scale of each tissue.

### 2.10. Statistical Analysis

The data were tested for normality and homogeneity using the Shapiro–Wilk and Levene tests. Parametric data are expressed as mean ± standard deviation (SD) and analyzed via two-way ANOVA. Here, we consider the effects of the following factors (F): Be-oil supplementation (Be), obesity (Ob), and interaction (I: Be × Ob). When F was significant, a post hoc Tukey test was applied. Non-parametric data are presented as median with interquartile range [IQR] through a generalized linear model (GLM) with the appropriate model selected based on the Akaike and Bayesian information criterion (AIC; BIC). Post hoc pairwise comparisons were performed using estimated marginal means (emmeans package) with Tukey adjustment. The significance level adopted was *p* < 0.05. The analyses were conducted using R Core Team software (2025), v4.5.2.

## 3. Results

### 3.1. Effects of Oral Be-Oil Supplementation on Body Weight Evolution, Food Intake (Figure 2), and Metabolism (Table 1) in Lean (CON) and Hypothalamic Obese (MSG) Male Wistar Rats

In males, BW in CON and MSG-obese animals progressively increased over time (PND 30–90) in all groups, throughout the experimental period (Figure 2A). However, ∆BW was significantly influenced by MSG treatment in the males (F_(1,35)_ = 69.86; *p* < 0.0001; Figure 2A), resulting in smaller AUC and decreased ∆BW in MSG_Ns_ (30.97%; *p* < 0.0001), in relation to CON_Ns_, and MSG_Be_ (27.83%; *p* < 0.0001) in relation to CON_Be_ groups (Figure 2B,C).

**Figure 2:**
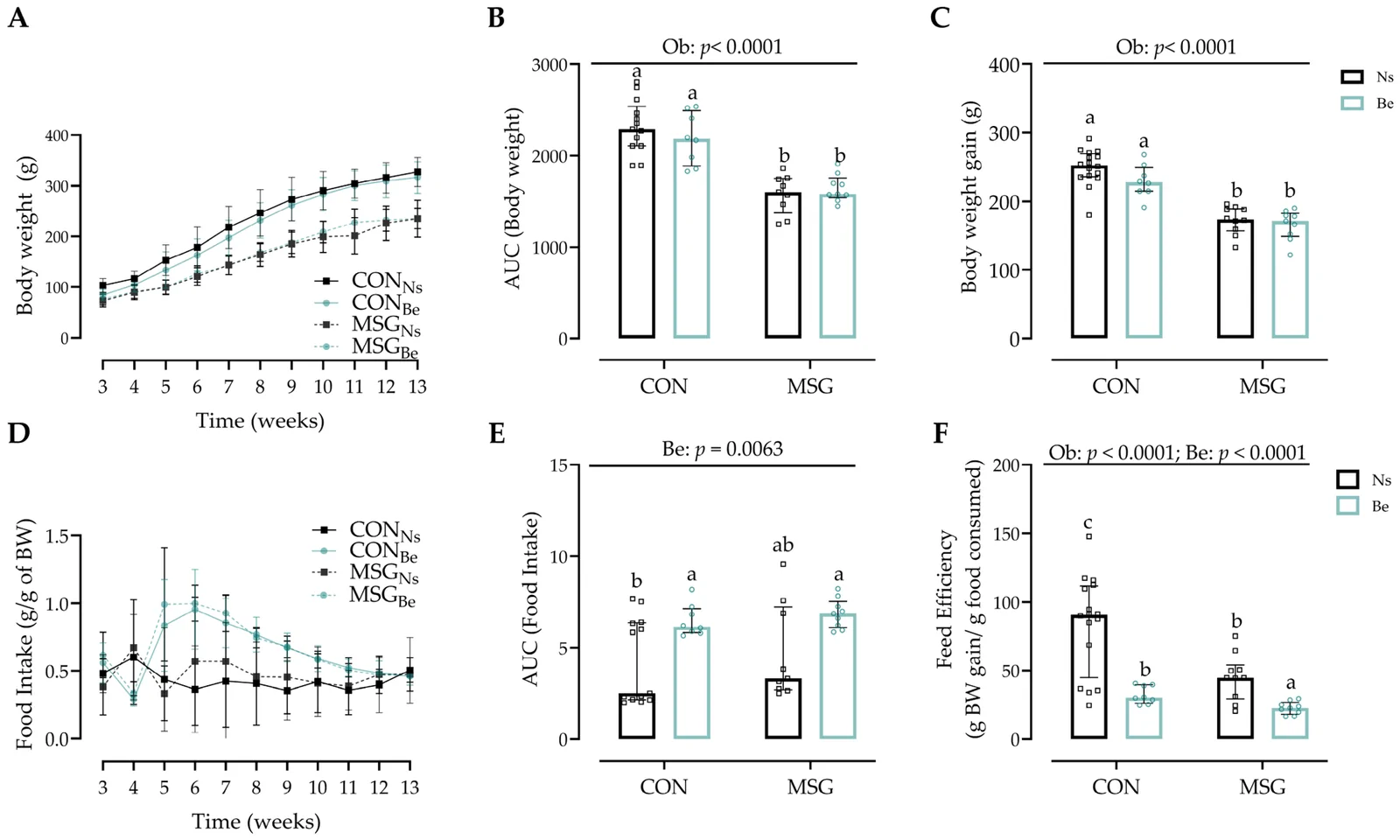
Effects of oral Be-oil supplementation on the body weight, food intake and feed efficiency rate over time in male CON (lean) and MSG-obese Wistar rats. (**A**) Body weight (weeks); (**B**) area under the curve (AUC) of body weight; (**C**) ∆BW; (**D**) food intake (weeks); (**E**) area under the curve (AUC) of food intake; (**F**) feed efficiency rate (FER). In Figure (**B**,**C**), data are expressed as mean ± SD (*n* = 8–10 rats per group). Two-way ANOVA with *p*-values of the factor (**F**) are indicated on lines above the graphs: Ob: obesity and Be: Be-oil supplementation and I (Ob vs. Be). Different letters above the bars indicate statistical differences between the groups (post hoc Tukey test; *p* < 0.05). In Figure (**E**,**F**), data are expressed as median with IQR. GLM. Groups that share at least one letter are not significantly different, while groups with different letters are significantly different.

In male rats, the food intake was influenced only by Be-oil supplementation (F_(1,36)_ = 8.44; *p* = 0.0063), resulting in higher food intake in CON_Be_ and MSG_Be_ compared with the respective Ns groups (Figure 2D,E). However, when FER was evaluated, we noted significant influences of Ob (F_(1,41)_ = 42.73; *p* < 0.0001) and Be-oil (F_(1,40)_ = 65.67; *p* < 0.0001). Thus, reduced FER was found in the MSG_Ns_ (46.29%; *p* < 0.0001); MSG_Be_ (72.88%; *p* < 0.0001) and CON_Be_ (61.37%; *p* < 0.0001) groups in comparison with CON_Ns_ rats (Figure 2F).

MSG treatment modified final BW (F_(1,39)_ = 67.47; *p* < 0.0001); naso-anal length (F_(1,39)_ = 84.26; *p* < 0.0001) and Lee index (F_(1,39)_ = 27.13; *p* < 0.0001). Thus, at PND 90 the MSG rats presented reduced BW (MSG_Ns_ 26.42%; *p* < 0.0001 and MSG_Be_: 21.84%; *p* = 0.0004) and naso-anal length (MSG_Ns_: 18.27%; *p* < 0.0001 and MSG_Be_: 13.68%; *p* = 0.0001) in relation to the respective CON_Ns_ and Be groups. In contrast, the Lee index was increased by 10.43% (*p* = 0.0002) in MSG_Ns_ when compared with CON_Ns_ and by 7.11% (*p* = 0.0292) in MSG_Be_ when compared with the CON_Be_ group (Table 1).

**Table 1:** Effects of oral Be-oil supplementation on body weight, plasma biochemical variables and insulin responsiveness in male lean and MSG-obese Wistar rats at PND90.

Male	Groups				*p*-Value (F)		
	CON_Ns_	CON_Be_	MSG_Ns_	MSG_Be_	Ob	Be	I
Final BW (g) *	323.75 ± 36.94 ^a^	309.63 ± 30.47 ^a^	238.20 ± 26.33 ^b^	242.00 ± 25.22 ^b^	<0.0001 (67.47)	0.568 (0.33)	0.371 (0.82)
Naso-anal length (cm) *	21.84 ± 1.42 ^a^	21.56 ± 0.50 ^a^	17.85 ± 1.20 ^b^	18.61 ± 1.43 ^b^	<0.0001 (84.26)	0.600 (0.28)	0.196 (1.73)
Lee index *	0.31 ± 0.02 ^c^	0.31 ± 0.01 ^bc^	0.35 ± 0.02 ^a^	0.34 ± 0.02 ^ab^	<0.0001 (27.13)	0.255 (1.34)	0.341 (0.93)
Total cholesterol (mg/dL) *	102.50 ± 13.48	90.41 ± 20.33	102.42 ± 18.11	101.45 ± 12.25	0.259 (1.33)	0.337 (0.95)	0.390 (0.76)
Triglyceride (mg/dL) *	57.82 ± 10.25 ^b^	76.45 ± 27.17 ^b^	82.95 ± 13.33 ^b^	139.48 ± 26.75 ^a^	<0.0001 (26.69)	0.0006 (15.99)	0.0508 (4.26)
Glucose (mg/dL) *	83.33 ± 2.66 ^b^	83.10 ± 8.25 ^b^	97.83 ± 6.88 ^a^	90.70 ± 7.16 ^ab^	0.0002 (17.25)	0.1570 (2.11)	0.1840 (1.855)
Insulin (ng/mL) ^†^	0.21 [0.07] ^a^	0.08 [0.17] ^a^	0.83 [0.17] ^b^	0.35 [0.16] ^a^	<0.0001 (25.73)	0.0016 (23.18)	0.0976 (2.93)
TyG Index *	7.77 ± 0.21 ^b^	7.98 ± 0.13 ^b^	8.30 ± 0.16 ^ab^	8.71 ± 0.26 ^a^	<0.0001 (27.87)	0.0229 (5.98)	0.4065 (0.71)
HOMA-IR ^†^	1.00 [0.38] ^a^	0.380 [0.91] ^a^	4.81 [0.32] ^b^	1.83 [1.01] ^a^	<0.0001 (30.42)	0.0009 (13.77)	0.0752 (3.41)

* Data expressed as mean ± SD (*n* = 10–20 rats per group). *p*-values of factor (F) effects obtained in the Two-way ANOVA are shown Ob: obesity; Be: Be-oil supplementation; I: interaction (Ob × Be). ^†^ Data expressed as median with [IQR]. Generalized linear model. *p*-value < 0.05. Different letters above the numbers indicate statistical differences between the groups (post hoc Tukey test; *p* < 0.05). Groups that share at least one letter are not significantly different, while groups with different letters are significantly different. CON: control (lean); MSG: monosodium glutamate (obese); Ns: non-supplemented; Be: Be-oil-supplemented.

Furthermore, hypothalamic MSG-obesity also altered fasting glucose levels (F_(1,28)_ = 17.25; *p* = 0.0002) with MSG_Ns_ male rats presenting increased glucose plasma levels in relation to males in the CON groups (MSG_Ns_ vs. CON_Ns_: 17.40%; *p* = 0.0059 and MSG_Ns_ vs. CON_Be_: 17.73%; *p* = 0.0016). Similarly, triglyceride levels were influenced by MSG-obesity (F_(1,22)_ = 26.69; *p* < 0.0001). However, effects of Be-oil supplementation (F_(1,22)_ = 15.99; *p* = 0.0006) and interactions (F_(1,22)_ = 4.99; *p* = 0.0508) were also noted for this parameter. Consequently, MSG_Be_ males had increased triglyceride levels in relation to the other groups (Table 1). Total cholesterol values were not altered by either MSG-obesity or Be-oil.

Circulating fasting insulin levels were also altered by MSG-obesity (F_(1,30)_ = 25.73; *p* < 0.0001), with MSG_Ns_ demonstrating hyperinsulinemia in relation to the other groups. Moreover, insulin was also modified by Be-oil (F_(1,29)_ = 23.18; *p* = 0.0016), resulting in a reduction of 80.62% in plasma insulin levels in the MSG_Be_ in comparison with MSG_Ns_ (Table 1).

The observed changes in fasting metabolic values resulted in significant influences on IR biomarkers. In this regard, MSG-obesity altered TyG index (F_(1,20)_ = 18.76; *p* = 0.0003) with slight increases found in MSG groups in comparison with their CON counterparts. Interestingly, Be-oil supplementation also modified the TyG index (F_(1,22)_ = 5.98; *p* = 0.0229) with MSG_Be_ presenting elevated TyG values in relation to other groups. Similarly, HOMA-IR was also influenced by MSG-obesity (F_(1,30)_ = 30.42; *p* < 0.0001) with MSG_Ns_ rats presenting elevated values (88.62%) in relation to the CON_Ns_ group. Moreover, for HOMA-IR we also noted an effect of Be-oil supplementation (F_(1,29)_ = 13.77; *p* = 0.0009). However, MSG_Be_ male rats displayed lower HOMA-IR values (61.95%; *p* = 0.0030) than those of the MSG_Ns_ group.

### 3.2. Effects of Oral Be-Oil Supplementation on Body Weight Evolution, Food Intake Consumption (Figure 3A–F) and Metabolism (Table 2) in Lean (CON) and Hypothalamic Obese (MSG) Female Wistar Rats

From PND 30 to 90, a rise in ∆BW was noted in all experimental groups (Figure 3A). A significant effect of MSG-obesity was found on the AUC for BW (F_(1,62)_ = 118.07; *p* < 0.0001) and ∆BW (F_(1,63)_ = 114.84; *p* < 0.0001). As a result, MSG_Ns_ and MSG_Be_ presented lower BW AUC (20.73% and 28.44% respectively) and ∆BW (28.39% and 26.59%) in comparison with females in the CON_Ns_ group (Figure 3B,C). Moreover, Be-oil also modified AUC of BW (F_(1,62)_ = 19.87; *p* < 0.0001), with CON_Be_ and MSG_Be_ groups presenting reductions of 9.52% (*p* = 0.0070) and 9.73% (*p* = 0.0219), respectively, in relation to the respective Ns rats.

**Figure 3:**
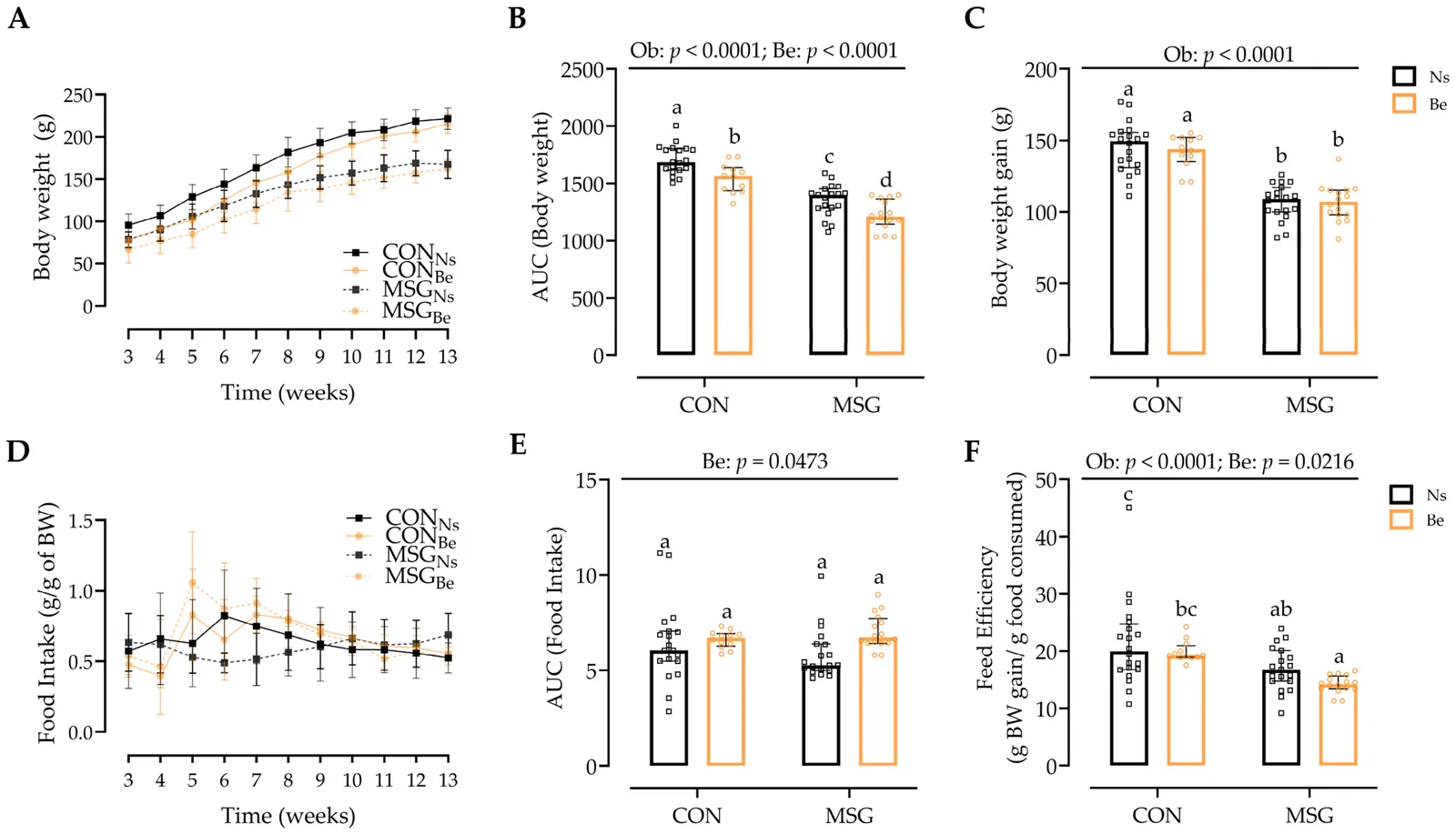
Effects of oral Be-oil supplementation on body weight, food intake and feed efficiency rate over time in female CON (lean) and MSG-obese Wistar rats. (**A**) Body weight (weeks); (**B**) area under the curve (AUC) of body weight; (**C**) ∆BW; (**D**) food intake (weeks); (**E**) area under the curve (AUC) of food intake; (**F**) feed efficiency rate (FER). In Figure (**B**,**C**), data are expressed as mean ± SD (*n* = 8–10 rats per group). Two-way ANOVA with *p*-values of the factor (**F**) are indicated on lines above the graphs: Ob: obesity; Be: Be-oil supplementation; I: interaction (Ob vs. Be). Different letters above the bars indicate statistical differences between the groups (post hoc Tukey test; *p* < 0.05). In Figure (**E**,**F**), data are expressed as median with IQR. GLM. Groups that share at least one letter are not significantly different, while groups with different letters are significantly different.

Food intake was influenced by Be-oil effects (F_(1,63)_ = 3.01; *p* = 0.0473), but without significance in the post hoc test (Figure 3E). FER was significantly altered by MSG-obesity (F_(1,65)_ = 25.76; *p* < 0.0001) and the MSG_Ns_ group displayed lower FER than CON_Ns_ rats (Figure 3F). However, Be-oil also modulated FER (F_(1,64)_ = 5.54; *p* = 0.0216), resulting in lower FER in the CON_Be_ (6.59%; *p* < 0.0001) and MSG_Be_ (15.98%; *p* < 0.0001) groups, compared with the respective Ns groups.

Only MSG-obesity influenced final BW (F_(1,39)_ = 67.47; *p* < 0.0001); naso-anal length (F_(1,39)_ = 84.26; *p* < 0.0001) and Lee index in females (F_(1,39)_ = 27.13; *p* < 0.0001) (Table 2). In this regard, MSG_Ns_ and MSG_Be_ females presented decreased BW (22.74%, *p* < 0.0001 and 20.59%, *p* < 0.0001, respectively) and naso-anal length (13.16%, *p* < 0.0001 and 15.79%, *p* < 0.0001, respectively), respectively, as well as an increased Lee index (6.25%, *p* < 0.0001 and 9.37%, *p* = 0.0019, respectively), when compared with their CON_Ns_ and CON_Be_ groups. Total cholesterol was influenced only by Be-oil (F_(1,20)_ = 8.31; *p* = 0.0092), with CON_Be_ rats showing lower (25.86%, *p* = 0.0115) plasma values of total cholesterol in comparison with the CON_Ns_ group. Fasting triglyceride levels were influenced by MSG-obesity (F_(1,22)_ = 26.69; *p* < 0.0001), with higher (69.91%, *p* = 0.0094) triglyceride levels found in MSG_Ns_ females than in CON_Ns_. Moreover, MSG_Be_ also showed elevated triglyceride levels (55.31%, *p* = 0.0142) compared with females in the CON_Be_ group. Neither MSG-obesity nor Be-oil supplementation altered fasting glucose levels (Table 2). However, plasma insulin levels were influenced by MSG-obesity (F_(1,31)_ = 8.47; *p* = 0.0068) and Be-oil supplementation (F_(1,30)_ = 8.53; *p* = 0.0067). Thus, higher insulin levels were found in MSG_Be_ in comparison with the CON_Ns_ (1750.00%; *p* = 0.0047), CON_Be_ (825.00%; *p* = 0.0494), and MSG_Ns_ (640.00%; *p* = 0.0175) groups.

**Table 2:** Effects of oral Be-oil supplementation on body weight, plasma biochemical variables and insulin responsiveness in female lean and MSG-obese Wistar rats at PND90.

Female	Groups				*p*-Value (F)		
	CON_Ns_	CON_Be_	MSG_Ns_	MSG_Be_	Ob	Be	I
Final BW (g) *	222.10 ± 15.86 ^a^	215.73 ± 17.11 ^a^	171.58 ± 14.72 ^b^	171.31 ± 10.37 ^b^	<0.0001 (180.50)	0.416 (0.67)	0.412 (0.68)
Naso-anal length (cm) ^†^	19.00 [1.50] ^a^	19.00 [0.75] ^a^	16.50 [1.00] ^b^	16.00 [0.62] ^b^	<0.0001 (153.98)	0.070 (3.39)	0.467 (0.54)
Lee index *	0.32 ± 0.02 ^a^	0.32 ± 0.01 ^a^	0.34 ± 0.01 ^b^	0.35 ± 0.01 ^b^	<0.0001 (41.87)	0.101 (2.77)	0.820 (0.05)
Total cholesterol (mg/dL) *	101.99 ± 18.20 ^a^	75.61± 9.92 ^b^	89.52 ± 10.69 ^ab^	94.07 ± 11.49 ^ab^	0.5243 (0.42)	0.0092 (8.31)	0.0541 (4.19)
Triglyceride (mg/dL) *	46.20 ± 3.71 ^bc^	44.15 ± 12.86 ^c^	78.50 ± 17.08 ^a^	68.57 ± 18.52 ^ab^	<0.0001 (23.92)	0.3310 (0.99)	0.5080 (0.45)
Glucose (mg/dL) *	96.00 ± 8.12 ^a^	93.90 ± 7.95 ^a^	100.28 ± 7.20 ^a^	96.20 ± 5.67 ^a^	0.212 (1.63)	0.231 (1.49)	0.702 (0.15)
Insulin (ng/mL) ^†^	0.02 [0.04] ^a^	0.04 [0.17] ^a^	0.05 [0.07] ^a^	0.37 [0.37] ^b^	0.0069 (8.47)	0.0067 (8.53)	0.2311 (1.49)
TyG Index *	7.67 ± 0.12 ^bc^	7.59± 0.27 ^c^	8.28 ± 0.21 ^a^	8.07 ± 0.28 ^ab^	<0.0001 (29.90)	0.164 (2.07)	0.528 (0.41)
HOMA-IR ^†^	0.11 [0.24] ^a^	0.24 [0.99] ^a^	0.27 [0.43] ^a^	2.00 [1.85] ^b^	0.0045 (9.49)	0.0054 (9.03)	0.2748 (1.24)

* Data expressed as mean ± SD (*n* = 10–20 rats per group). *p*-values of factor (F) effects obtained in the Two-way ANOVA are shown: Ob: obesity; Be: Be-oil supplementation; I: interaction (Ob × Be). ^†^ Data expressed as median with [IQR]. GLM. *p*-value < 0.05. Different letters above the numbers indicate statistical differences between the groups on the post hoc Tukey test (*p* < 0.05). Groups that share at least one letter are not significantly different, while groups with different letters are significantly different. CON: control (lean); MSG: monosodium glutamate (obese); Ns: non-supplemented; Be: Be-oil-supplemented.

Due to effects on fasting metabolic parameters, the IR biomarker indices were also altered in females. Thus, MSG-obesity influenced the TyG (F_(1,22)_ = 27.41; *p* < 0.0001) and HOMA-IR (F_(1,31)_ = 9.49; *p* = 0.0045) indices. Therefore, females in the MSG_Ns_ and MSG_Be_ groups presented higher (6.59%, *p* = 0.0093; 6.15%, *p* = 0.0054) TyG values than those of the CON animals. Similarly, as a consequence of the Be-oil effect (F_(1,30)_ = 9.03; *p* = 0.0054), MSG_Be_ had higher HOMA-IR, in relation to the other groups (Table 2).

### 3.3. Adiposity and Histological Modifications Induced by Be-Oil Supplementation in WAT and BAT of Lean (CON) and Hypothalamic Obese (MSG) Male Wistar Rats

MSG-obesity exerted an influence on WAT-I mass (F_(1,38)_ = 94.18; *p* < 0.0001), adipocyte area (F_(1,16)_ = 67.69; *p* < 0.0001), and number (F_(1,16)_ = 42.40; *p* < 0.0001). Thus, WAT-I mass was 159.23% and 256.97% higher, respectively, in the MSG_Ns_ and MSG_Be_ animals in relation to their respective CON groups (*p* < 0.0001). Similarly, the MSG groups had hypertrophic adipocytes (MSG_Ns_ vs. CON_Ns_: 63.50%; *p* = 0.0002; MSG_Be_ vs. CON_Be_: 66.26%; *p* = 0.0001) and lower adipocyte numbers (MSG_Ns_ vs. CON_Ns_: 126.63%; *p* = 0.0034; MSG_Be_ vs. CON_Be_: 136.61%; *p* = 0.0007), in relation to the respective lean groups (Figure 4A–C). MSG-obesity also altered WAT-P mass (F_(1,39)_ = 47.44; *p* < 0.0001) and adipocyte number (F_(1,16)_ = 10.25; *p* = 0.0056), without changing the WAT-P area (F_(1,16)_ = 3.93; *p* = 0.0648). Thus, MSG animals (Ns and Be) presented elevated WAT-P mass (MSG_Ns_ vs. CON_Ns_: 117.92%; *p* < 0.0001; MSG_Be_ vs. CON_Be_: 172.40%; *p* = 0.0259), compared with CON_Ns_ with reduced adipocyte number (although this was not significant in Tukey’s post hoc test) (Figure 4D–F). Representative photomicrographs of these data are shown in Figure 4J–Q.

The iBAT depot mass of male rats was also influenced by MSG-obesity (F_(1,22)_ = 29.51; *p* < 0.0001), as demonstrated by a significant interaction (Be vs. Ob; F_(1,22)_ = 8.90; *p* = 0.0068). Consequently, iBAT content was significantly higher in the MSG_Ns_ (82.10%; *p* < 0.0001) and MSG_Be_ (57.24%; *p* = 0.0057) groups than in CON_Ns_ rats (Figure 4G). Moreover, CON_Be_ male rats had an iBAT mass that was similar to that of MSG_Be_ animals. MSG-obesity also modified adipocyte number (F_(1,18)_ = 14.69; *p* = 0.0012) and area (F_(1,18)_ = 40.04; *p* < 0.0001) in male rats. For this reason, augmented area (MSG_Ns_ vs. CON_Ns_: 63.61%; *p* = 0.0049; MSG_Be_ vs. CON_Be_: 90.83%; *p* = 0.0003) and reduced nuclei number (MSG_Ns_ vs. CON_Ns_: 34.77%; *p* = 0.0260; MSG_Be_ vs. CON_Be_: 24.50%; *p* = 0.0170) were observed in iBAT from MSG groups, in relation to iBAT from CON rats (Figure 4H,I; photomicrographs: Figure 4R–U).

### 3.4. Adiposity and Histological Modifications Induced by Be-Oil Supplementation in WAT and BAT of Lean (CON) and Hypothalamic Obese (MSG) Female Wistar Rats

MSG-obesity altered WAT-I mass (F_(1,62)_ = 80.63; *p* < 0.0001), with effects on adipocyte area (F_(1,16)_ = 150.25; *p* < 0.0001) and number (F_(1,16)_ = 252.81; *p* < 0.0001) (Figure 5A–C). As a consequence, higher WAT-I mass (MSG_Ns_ vs. CON_Ns_: 356.25%; *p* < 0.0001; MSG_Be_ vs. CON_Be_: 608.15%; *p* = 0.0259), adipocyte hypertrophy (MSG_Ns_ vs. CON_Ns_: 205.87%; *p* < 0.0001 MSG_Be_ vs. CON_Be_: 173.70%; *p* < 0.0001), and reductions in adipocyte number (MSG_Ns_ vs. CON_Ns_: 77.15%; *p* < 0.0001; MSG_Be_ vs. CON_Be_: 71.78%; *p* < 0.0001) were noted in the MSG groups compared with the same variables in the respective lean (CON) rats (Figure 5J–M).

Similarly, MSG-obesity also modified WAT-P mass (F_(1,62)_ = 62.92; *p* < 0.0001), influencing the adipocyte area (F_(1,16)_ = 40.25; *p* < 0.0001) and number (F_(1,16)_ = 53.66; *p* < 0.0001) in this depot. As a result, WAT-P weight was significantly augmented in MSG_Ns_ (109.23%; *p* < 0.0001) and MSG_Be_ (164.46%; *p* < 0.0001) females, in relation to the respective CON rats (Figure 5D). Moreover, smaller nuclei numbers (MSG_Ns_: 74.17%; *p* < 0.0001 and MSG_Be_: 60.83%; *p* = 0.0023) were observed in WAT-P, when compared with the respective CON_Ns_ animals (Figure 5E). In WAT-P from MSG_Ns_, hypertrophic adipocytes (158.08%; *p* < 0.0001) were also observed, in relation to CON_Ns_. However, in the WAT-P depot, we found a significant interaction (Be vs. Ob) between the number (F_(1,62)_ = 10.65; *p* = 0.0049) and area (F_(1,16)_ = 26.97; *p* < 0.0001) of adipocytes. Here, we also noted a Be-oil supplementation effect on adipocyte area (F_(1,16)_ = 16.93; *p* = 0.0008). Consequently, the WAT-P of the MSG_Be_ female group displayed a smaller adipocyte area (49.41%; *p* < 0.0001) and an increase in adipocyte number (51.61%; *p* = 0.0152), in relation to the MSG_Ns_ animals (Figure 5E,F). These results can also be observed in representative photomicrographs in Figure 5N–Q.

In the iBAT depot from females, we also found a significant influence of MSG-obesity on mass (F_(1,23)_ = 59.73; *p* < 0.0001), adipocyte area (F_(1,20)_ = 5.06; *p* = 0.0359), and number (F_(1, 20)_ = 93.63; *p* < 0.0001), compared with the CON groups (Figure 5G–I). Similarly, the mass was significantly increased in iBAT from MSG groups in comparison with respective CON animals (MSG_Ns_ vs. CON_Ns_: 77.42%; *p* = 0.0001; MSG_Be_ vs. CON_Be_: 71.24%; *p* < 0.0001), and the number of nuclei was significantly lower in iBAT (MSG_Ns_ vs. CON_Ns_: 48.33%; *p* = 0.0001; MSG_Be_ vs. CON_Be_: 59.35%; *p* < 0.0001). Representative photomicrographs of the iBAT are shown in the Figure 5R–U. With regard to the iBAT adipocyte area in female rats, no significant differences were observed among the experimental groups in the post hoc analysis.

### 3.5. Impact of Be-Oil Supplementation on Glucose Homeostasis and Islet Histology in Lean (CON) and Hypothalamic Obese (MSG) Male Wistar Rats

The oGTT and its respective curve pattern are shown in Figure 6A–C. After the oral glucose load, all groups presented augmented values of glycemia which returned to basal values over the time (Figure 6A). Interaction effects between Ob and Be-oil (F_(1,26)_ = 10.02; *p* = 0.0039) were noted in the AUC of oGTT with the MSG_Be_ group presenting increased AUC in comparison with CON_Be_ (*p* = 0.0090) and MSG_Ns_ (*p* = 0.0190) (Figure 6A insert). The time of the oGTT peak was modified by Be-oil supplementation (F_(1,27)_ = 4.36; *p* = 0.0467), and MSG_Be_ animals reached peak glucose levels more quickly than CON_Be_ rats (Figure 6B). Moreover, glucose peak magnification was modified by the factors Ob (F_(1,26)_ = 6.96; *p* = 0.0138) and Be-oil supplementation (F_(1,26)_ = 7.32; *p* = 0.0118), as well as by the interaction Be vs. Ob (F_(1,26)_ = 18.86; *p* = 0.0002; Figure 6C). Thus, MSG_Be_ rats presented an augmented glucose peak value compared with other groups (MSG_Be_ vs. CON_Ns_: 21.27%; *p* = 0.0130); MSG_Be_ vs. CON_Be_: 29.58%; *p* = 0.0002 and MSG_Be_ vs. MSG_Ns_: 36.11%; *p* = 0.0001). Neither Ob nor Be-oil supplementation significantly interfered with GIIS (Figure 6D). However, Ob effects were noted on the area (F_(1,21)_ = 14.04; *p* = 0.0012) and number (F_(1,21)_ = 6.04; *p* = 0.0227) of pancreatic islets in the pancreas. As such, there was a higher number of islets (74.71%; *p* = 0.0301) in the endocrine pancreas of MSG_Ns_ rats than in the endocrine pancreas of CON_Ns_ (Figure 6E). Moreover, the islet area was significantly smaller in the MSG_Be_ endocrine pancreas (52.36%; *p* = 0.0049) in relation to that of CON_Be_ rats. An interaction of Be-oil (F_(1,21)_ = 7.86; *p* = 0.0106; Be vs. Ob) (F_(1,21)_ = 4.75; *p* = 0.0407) was observed for islet number, resulting in a reduced islet number in the pancreas of MSG_Be_ rats in relation to MSG_Ns_. These histological effects may be observed in the representative photomicrographs shown in Figure 6G–J. HOMA-β was not significantly altered by either MSG-obesity or Be-oil supplementation (Table S2).

### 3.6. Impact of Be-Oil Supplementation on Glucose Homeostasis and Islet Histology in Lean (CON) and Hypothalamic Obese (MSG) Female Wistar Rats

In all experimental groups, we observed augmented glycemia values immediately after the glucose load; however, no significant difference was observed in the AUC during oGTT (Figure 7A). Moreover, MSG-obesity modified the glucose peak (F_(1,29)_ = 5.52; *p* = 0.0258; Figure 7C), but without a significant effect in the post hoc test. Neither Ob nor Be-oil supplementation showed significant effects on time to glucose peak during oGTT in females (Figure 7B). Similarly, islet number was not influenced by either MSG-obesity or Be-oil supplementation. However, MSG-obesity altered GIIS (F_(1,38)_ = 43.42; *p* = 0.0037), the area of pancreatic islets (F_(1,18)_ = 11.89; *p* = 0.0033) and HOMA-β (F_(1,31)_ = 5.42; *p* = 0.0270). Thus, MSG_Ns_ females had a significant reduction in pancreatic islet area (78.58%; *p* = 0.0075), while MSG_Be_ had a significant reduction in GIIS (25.75%; *p* = 0.0165; Figure 7D) and augmented HOMA-β (59.73%; *p* = 0.0190; Table S2), in comparison with the CON_Ns_ group.

## 4. Discussion

In the present study, neonatally MSG-treated males and females developed obesity in adulthood, characterized by elevated WAT and iBAT mass, accompanied by adipocyte hypertrophy, and associated with metabolic dysfunctions and IR confirming data previously published by us [51,52] and others [53,54]. However, we noted that, in the MSG-obese females, these fasting-state alterations were less marked than those observed in MSG-obese males. Sex differences in MSG-treatment have been registered in other studies, with a worse metabolic state in MSG-obese males [31]. Autonomic imbalance, e.g., parasympathetic hyperactivity and sympathetic hypofunction, is a hallmark of MSG-induced obesity and has a central role in metabolic and adiposity abnormalities registered in this obese model [22,55]. Thus, elevated vagus nerve tone (vagal hyperactivity) is intimately linked to hyperinsulinemia in MSG-obese rats [55]. Augmented insulin levels lead to lipogenesis and adipogenesis in WAT and, over time, IR [22]. In contrast, the lower sympathetic flux leads to reduced thermogenesis into iBAT contributing to its hypofunction and the lower energy expenditure observed in the MSG-obese rodents [53]. Here, we also demonstrated that MSG-obese males and females were smaller and lighter in relation to their lean counterparts. In this regard, it is well-recognized that MSG-obese animals have reduced growth, skeletal and muscle mass and infertility, as well as hypercortisolism [36,54,56]. Moreover, we showed herein that MSG females and males did not develop hyperphagia; a condition commonly presented in diet-induced and genetic obese models in rodents [25,57].

Despite extensive hypothalamic lesions, MSG-obese animals are responsive to several anti-adiposity interventions, such as exercise training [24,58], amino acid supplementation [23,59], surgical and pharmacological strategies [25,60] and bioactive plant products [51,61]. In this regard, it is well established that Be in seeds or oils are excellent sources of lipids, vitamins, and several minerals that contribute to satiety and dietary satisfaction, adiposity reduction, and protection against weight gain [4,7,15,62]. To our knowledge, this is the first time that the long-term oral impact of Be-oil supplementation has been studied in the MSG-obese animals, considering sex influences.

Here we noted that, in both MSG-obese males and females, oral consumption of the Be-oil led to hyperphagia without body weight or adiposity gain, resulting in lower feed efficiency. Accordingly, obese female adolescents consuming Brazil nuts (15–25 g/day for 16 weeks) did not display any alterations in adiposity or BMI [62]. Similarly, men and women who ingested Brazil nuts daily for six months did not present changes in adiposity or BMI [63]. Thus, as suggested by Silva et al. [64], Brazil nut consumption cannot modulate weight when consumed in the context of a habitual diet, without energy restriction [64].

Many of the positive effects of Brazil nut consumption (in oil or seeds) on obesity and metabolic diseases have been attributed to its elevated levels of MUFA and PUFA [10,38]. According to analyses performed by Muniz et al. [65], the Be-oils used in the present study present a high content of MUFA oleic acid (38.5%) and of the PUFA linoleic acid (31.3%). However, this analysis also shows that Be-oil contains a significant percentage of saturated fatty acids, such as palmitic acid (14.3%) [65]. Central nervous system exposure to palmitic acid via direct infusion or by oral gavage was associated with impaired hypothalamic insulin and leptin signaling [66]. Insulin and leptin are important satiety signals at the hypothalamic level and central resistance to these hormones induces hyperphagia [66]. Interestingly, at the hypothalamic level mentioned, these effects on IR were independent of WAT, as also noted here. Thus, it is possible that the overeating found in the present study is related to the central effects of palmitic acid, since these authors also demonstrated that oleic acid does not lead to this effect.

In contrast, male obese mice supplemented with Be-oil (extracted from seeds) for 40 days demonstrated lower WAT and BW gain without altered food intake [67]. According to these authors, a high percentage of oleic and linoleic acids found in Be-oil may be responsible for the anti-obesity effects observed [67]. Here, we found a slight reduction in adipocyte hypertrophy in WAT-P, a visceral depot, of the MSG-obese females, without effects on MSG-obese males. Interestingly, also in the Be-oil-supplemented MSG-obese females, we noted a slight reduction in the AUC of BW. Thus, it appears that oral Be-oil consumption has greater anti-adiposity effects in MSG-obese females. Accordingly, MSG-obese females were more responsive to the anti-adiposity effects of prebiotic supplementation than MSG-obese males [68].

It is important to note that the overfeeding behavior observed, herein, in the Be-oil-supplemented groups was not associated with any gains in BW or adiposity, resulting in reduced feed efficiency as aforementioned. Greater energy expenditure, such as those caused by elevated thermogenesis could explain this finding. According to Falcone et al. [38], the anti-adiposity effect of refined Be-oil in the offspring could be related to elevated PUFA content and activation of uncoupling proteins in the mitochondria, which increases calorie burning and reduces fat storage [38]. In contrast, in the present study, the weight mass and adipocytes size in the well-recognized sites of thermogenesis (subcutaneous WAT-I and iBAT depots) were not altered by Be-oil chronic supplementation, in either male or female MSG-obese animals. Augmented muscular thermogenesis could also justify the reduced feed efficiency observed in the Be-oil-supplemented groups. In this regard, high PUFA reduced feed efficiency in high-fat induced obese male mice, thereby modulating mitochondrial function and efficiency in the skeletal muscle [69]. Further studies are necessary to evaluate these mechanisms in Be-oil-supplemented rats.

Obesity is frequently associated with glucose and lipid abnormalities, which lead to a high risk of diabetes and cardiovascular disease development [1]. Several studies in humans of both sexes have demonstrated that these metabolic abnormalities could be attenuated by the regular ingestion of Be, in the form of oil or seeds [10,63,70]. Few studies have explored these effects on rodents. In the present study, the oral glucose tolerance test (oGTT) and measurement of insulin responsiveness (TyG and HOMA-IR) were used to evaluate glucose homeostasis in male and female Be-oil-supplemented rats. MSG Be-oil-supplemented male rats presented an elevated glucose AUC, a higher glucose peak during oGTT, and an earlier glucose peak, in comparison with MSG_Ns_ male rats. In humans, a delayed glucose peak during oGTT is used as physiological indicator of prediabetes [71,72]. Thus, our oGTT data suggested improved insulin responsiveness. Consistent with this observation, we also found reduced HOMA-IR in these MSG-obese Be-oil-supplemented male rats. Thus, it appears that chronic oral Be-oil supplementation favors glycemic control in MSG-obese males, without altering fasting glucose values. In contrast, we noted that MSG-obese Be-oil-supplemented females presented an augmented HOMA-IR index, due to elevated fasting of insulin plasma levels, but this effect was not associated with significant changes in oral glucose tolerance. Be-oil is rich in Se, and in an observational study in humans, an increase in Se was associated with a 1.5% increase in insulin and a 1.7% increase in HOMA-IR [73]. As demonstrated below, Se exerts sex-dependent effects on health.

It is well recognized that sexual dimorphism influences glucose and lipid homeostasis in both mice and humans [74,75]. In the MSG-obese model, higher adiposity and a worsened pro-inflammatory state in males than females have been demonstrated [31]. Gao et al. [75] demonstrated that worsened glucose homeostasis in males is related to the profile of the gut microbiome, linking androgen and amino acid intestinal metabolism [75]. As mentioned earlier in this discussion, probiotics have more positive effects in MSG-obese females in comparison to MSG-obese males [68]. Thus, it is possible that several of the effects of Be-oil supplementation on glucose homeostasis, observed in the present work, involve sex influence effects.

In addition to their elevated MUFA and PUFA content, Brazil nuts are also rich in several micronutrients, particularly Se, which have been associated with many of their positive effects on health [76]. Enzymes involved in Se metabolism have been shown to act on glucose pathways with differences between sex [77]. However, contrasting effects of Brazil nuts and Se have been observed in glucose homeostasis. For example, non-diabetic subjects of both sexes, supplemented with 200 µg of Se for six weeks, displayed reduced glycated hemoglobin, but no alteration in fasting glucose [78]. In contrast, although the consumption of Brazil nuts for six months, in men and women, significantly elevated circulating levels of Se, with positive effects on the antioxidant system, fasting blood glucose significantly increased without any improvement in glycated hemoglobin [63]. On the other hand, it has also been demonstrated that Brazil nut consumption (1 seed for 8 week) decreased fasting glucose levels in healthy subjects of both sexes, a response that was related to selenoprotein polymorphisms [79]. Moreover, the addition of Brazil nuts to the diet of obese female adolescents elevates Se without significantly affecting glucose homeostasis (glucose, insulin and HOMA-IR), despite positive effects on the lipid profile [62]. Interestingly, selenocysteine lyase (Scly) catalyzes the hydrolysis of selenocysteine to selenide and alanine, facilitating re-utilization of Se for de novo selenoprotein synthesis. Scly^−/−^ mice display increased body weight and body fat composition, as well as altered lipid and carbohydrate metabolism, compared with wild-type mice. Additionally, male Scly^−/−^ mice are susceptible to developing glucose intolerance, whereas female Scly^−/−^ mice are protected [80].

The consumption of Brazil nuts has been associated with benefits to lipid metabolism in both sexes [74]. In the present work, we evaluated fasting values of triglycerides and total cholesterol in Be-oil-supplemented male and female rats. We noted that the effects of Be-oil supplementation on fasting triglycerides were different between male and females. Thus, MSG-obese males supplemented with Be-oil presented augmented triglyceride values, resulting an increased TyG index, which is a marker of IR. Similarly, daily supplementation of Be-oil for (10 mL) 30 days increased triglycerides in subjects with MS [10]. In contrast, in Be-oil-supplemented MSG-obese females, we noted a non-significant reduction in TyG, due to a slight reduction in triglycerides. Diminished triglycerides levels have also been reported in women and girls who consumed Brazil nuts [62,81]. On the other hand, patients of both sexes with hypertension and dyslipidemia who were supplemented with Be (13 g/day for 12 weeks) did not present any improvement in triglycerides [70]. Similar results were obtained in healthy subjects of both sexes [34].

In the present study, no significant effects of Be-oil supplementation were observed on total cholesterol in males, while a slight influence was observed in females. Donadio et al. [79] demonstrated that subjects (men and women) who consumed one Brazil nut a day for 8 weeks had reduced total cholesterol and glucose levels without any effects on triglycerides, HDL-c or LDL-c subfractions. According to these authors, the positive effect of Brazil nuts on lipids may be related to the presence of Se, particularly selenoproteins [79]. However, an important systematic review found adverse effects of Brazil nuts on lipid homeostasis [82], and a deeper evaluation is needed.

Insulin exerts a central role in lipid metabolism [1]. Thus, it is important to note that, in the present work, Be-oil caused opposing responses in fasting insulin levels, with MSG Be-oil-supplemented males presenting reductions while Be-oil-supplemented MSG females showed increased levels. Our pancreatic histological analysis indicates a reduced islet number in the pancreas of Be-oil-supplemented MSG males, where this response was not observed in Be-oil-supplemented MSG females. These data indicate that Be-oil may negatively impact islet mass in males. As mentioned previously, Be-oil is rich in palmitic and oleic acids, which both have well-recognized effects on insulin secretion and beta-cell mass [83]. Prolonged exposure to palmitate impairs β-cell function and mass [84]. Accordingly, palmitic and oleic acids modulate triglyceride pathways cultured β cells (line 1.1B4), altering apoptosis pathways at this target [85]. Thus, we hypothesized that chronic Be-oil supplementation in MSG males could result in high triglyceride accumulation in the endocrine pancreas, leading to a reduction in β-cell mass. Although non-significant, we also observed that islets from MSG Be-oil-supplemented male rats had lower GIIS.

This study has potential limitations. First, in the present study, we did not assess specific compounds present in the Be-oil that was offered to animals. As shown in Supplementary Table S1, the commercial Be-oil used here presents higher levels of MUFA and PUFA than those observed in several studies [10,86]. Moreover, the Se content of the Be-oil used in the present work is not known. However, using a similar extraction method applied to the AmazonOil company, Zhanglin et al. [40] confirmed that the high Se content in Be-oil (Table S1) is below toxic levels. Thus, the data suggest that the commercial Be-oil administered herein presents the principal bioactive compounds that have been commonly found in several studies using different analyses [40,87].

Secondly, sex hormones should also have been measured. There is no study exploring sexual hormones and Be-oil supplementation in rodents or humans, but PUFAs found in high concentration in this oil appear to be able to modulate estradiol and testosterone levels in other species [88,89,90]. Moreover, the estrous cycle in females can modulate metabolism [91,92]. However, given that estrus synchronization is common when females are grouped together, that the estrous cycle is short (4-5 days) in rats and that all the females of the subgroups were euthanized simultaneously, we believe that this aspect had little impact on the variable analyzed.

Finally, HOMA-IR and TyG index are common surrogate markers of IR, but their impact can be altered by age and sex [93]. Moreover, according to Kim et al. [93], TyG index, but not HOMA-IR, has emerged as a predictor of cardiovascular risk. The contrasting effects of Be-oil supplementation on IR in MSG-obese males and females observed here highlights the need to investigate these mechanisms. As such, further studies are necessary to clarify the impact of Be-oil supplementation on sexual hormone levels and their action.

## 5. Conclusions

We demonstrated herein that, in both sexes, the long-term oral consumption of Be-oil did not prevent hypothalamic obesity development. Moreover, independently of obesity or sex, Be-oil consumption led to reduced feed efficiency, suggesting an effect on energy homeostasis unrelated to changes in adiposity mass. Importantly, in MSG-obese rats, long-term oral Be-oil supplementation showed sex-dependent effects on triglycerides, insulin levels, glucose homeostasis, and endocrine pancreas, with more pronounced effects on MSG-male rats.

## Figures and Tables

**Figure 1:**
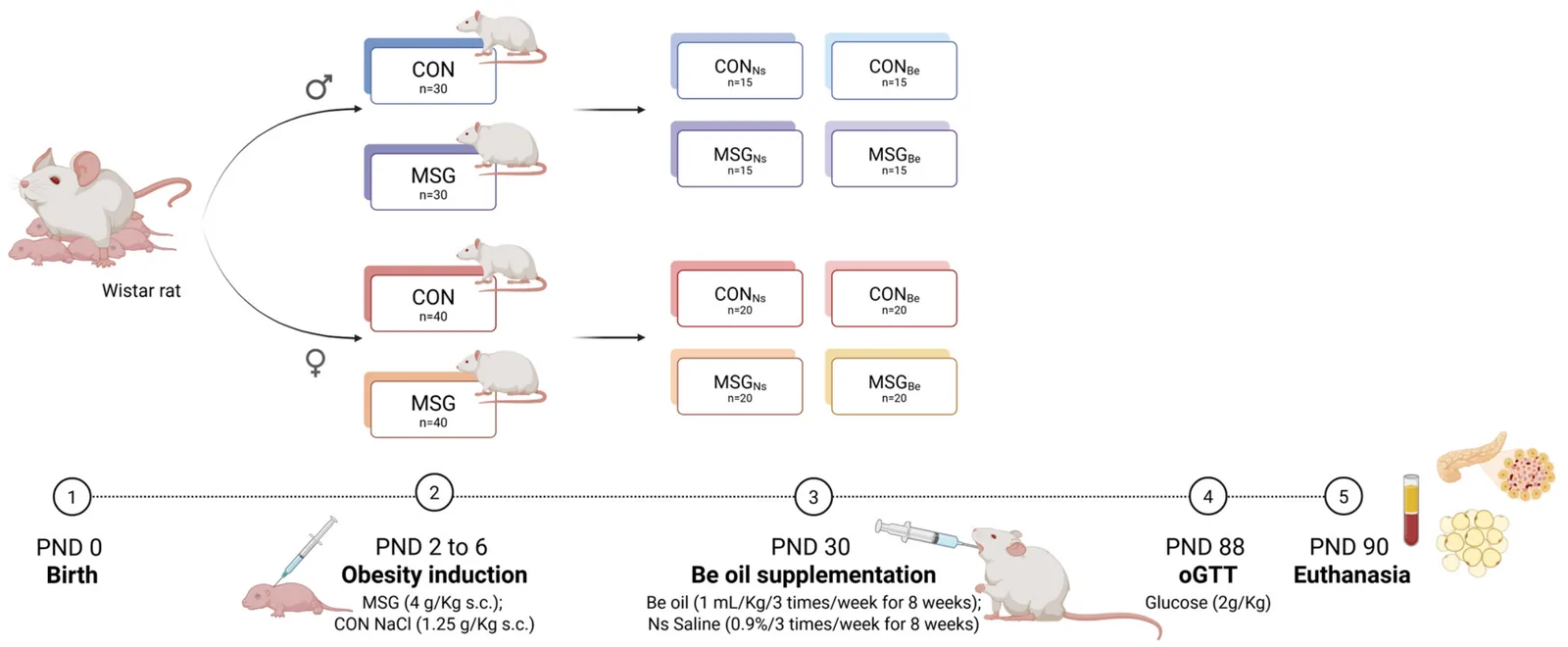
Experimental design. ♂: male Wistar rats; ♀: female Wistar rats; CON: control groups; MSG: hypothalamic obesity induced groups. Ns: non-supplemented rats; Be: Be-oil-supplemented rats; PND: postnatal day. Created in BioRender. Zawoski gomes, E. C. (2026) https://BioRender.com/9i6n72a (accessed on 7 July 2026).

**Figure 4:**
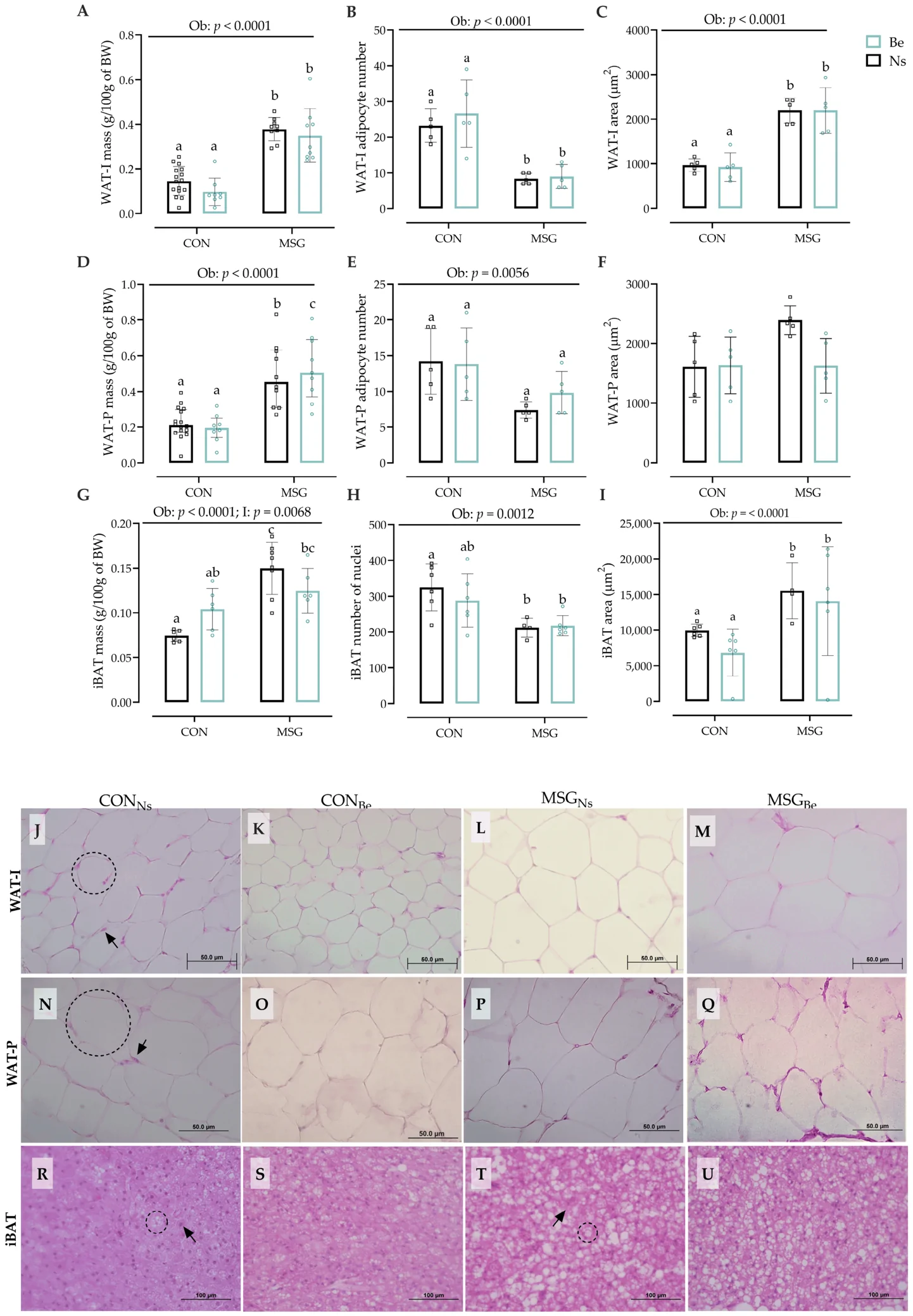
Effects of oral Be-oil supplementation on the WAT (P and I) and iBAT mass and histology in male Wistar rats. (**A**) WAT-I mass (g/100 g of BWt); (**B**) WAT-I adipocyte number; (**C**) WAT-I adipocyte area (µm^2^); (**D**) WAT-P mass (g/100 g of BW); (**E**) WAT-P l adipocyte number; (**F**) WAT-P adipocyte area (µm^2^); (**G**) iBAT mass (g/100 g of BW); (**H**) iBAT number nuclei; (**I**) iBAT adipocyte area (µm^2^). In Figure (**A**–**C**,**E**–**H**), data are expressed as mean ± SD (*n* = 5–6 rats per group). Two-way ANOVA with *p*-values of the factor (**F**) are indicated on the lines above the graphs: Ob: obesity; Be: Be-oil supplementation and I (Ob vs. Be). Different letters above the bars indicate statistical differences between the groups in the post hoc Tukey test (*p* < 0.05). Groups that share at least one letter are not significantly different, while groups with different letters are significantly different. In Figure (**D**), data are expressed as median with IQR. GLM. Representative photomicrographs showing H&E-stained: (**J**–**M**) WAT-I, Scale 50 μm; 40×; (**N**–**Q**) WAT-P, Scale 50 μm; 40×; (**R**–**U**) iBAT, scale 100 μm; 40×. Black arrows (−►) indicate adipocyte nuclei; and dotted circles indicate WAT and iBAT adipocytes.

**Figure 5:**
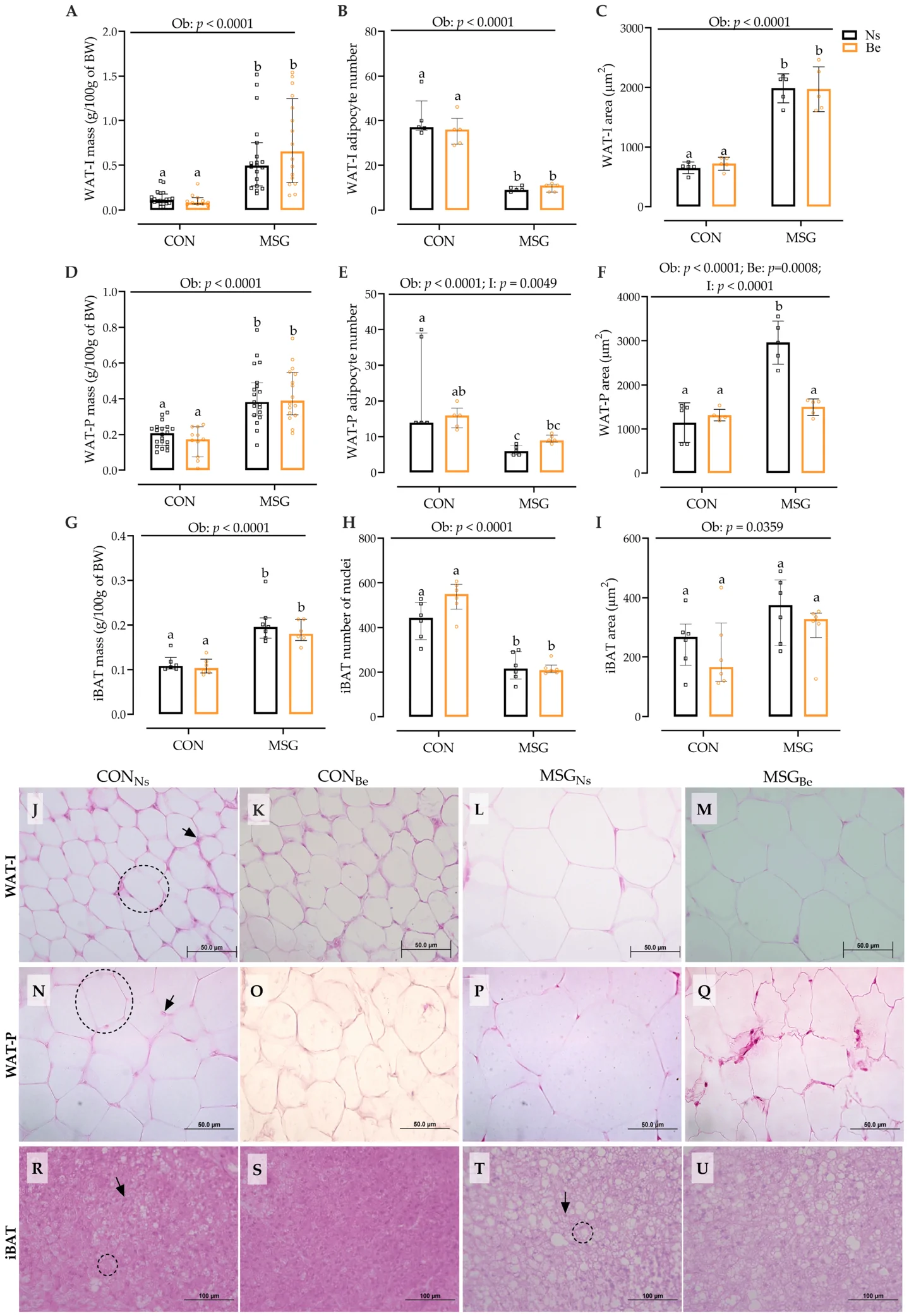
Effects of oral Be-oil supplementation on the WAT (P and I) and iBAT mass and histology of female Wistar rats. (**A**) WAT-I mass (g/100 g of BW); (**B**) WAT-I adipocyte number; (**C**) WAT-I adipocyte area (µm^2^); (**D**) WAT-P mass (g/100 g of BW); (**E**) WAT-P l adipocyte number; (**F**) WAT-P adipocyte area (µm^2^); (**G**) iBAT mass (g/100 g of BW); (**H**) iBAT number nuclei; (**I**) iBAT adipocyte area (µm^2^). In Figure (**C**,**F**,**H**,**I**), data are expressed as mean ± SD (*n* = 5–6 rats per group). Two-way ANOVA with *p*-values of the factor (F) are indicated on the lines above the graphs: Ob: obesity; Be: Be-oil supplementation; I: interaction (Ob vs. Be). Different letters above the bars indicate statistical differences between the groups in the post hoc Tukey test (*p* < 0.05). Groups that share at least one letter are not significantly different, while groups with different letters are significantly different. In Figure (**A**,**B**,**D**,**E**,**G**), data are expressed as median with IQR. GLM. Representative photomicrographs showing H&E-stained: (**J**–**M**) WAT-I, Scale 50 μm; 40×; (**N**–**Q**) WAT-P, Scale 50 μm; 40×; (**R**–**U**) iBAT, scale 100 μm; 40×. Black arrows (−►) indicate adipocyte nuclei; and dotted circles indicate WAT and iBAT adipocytes.

**Figure 6:**
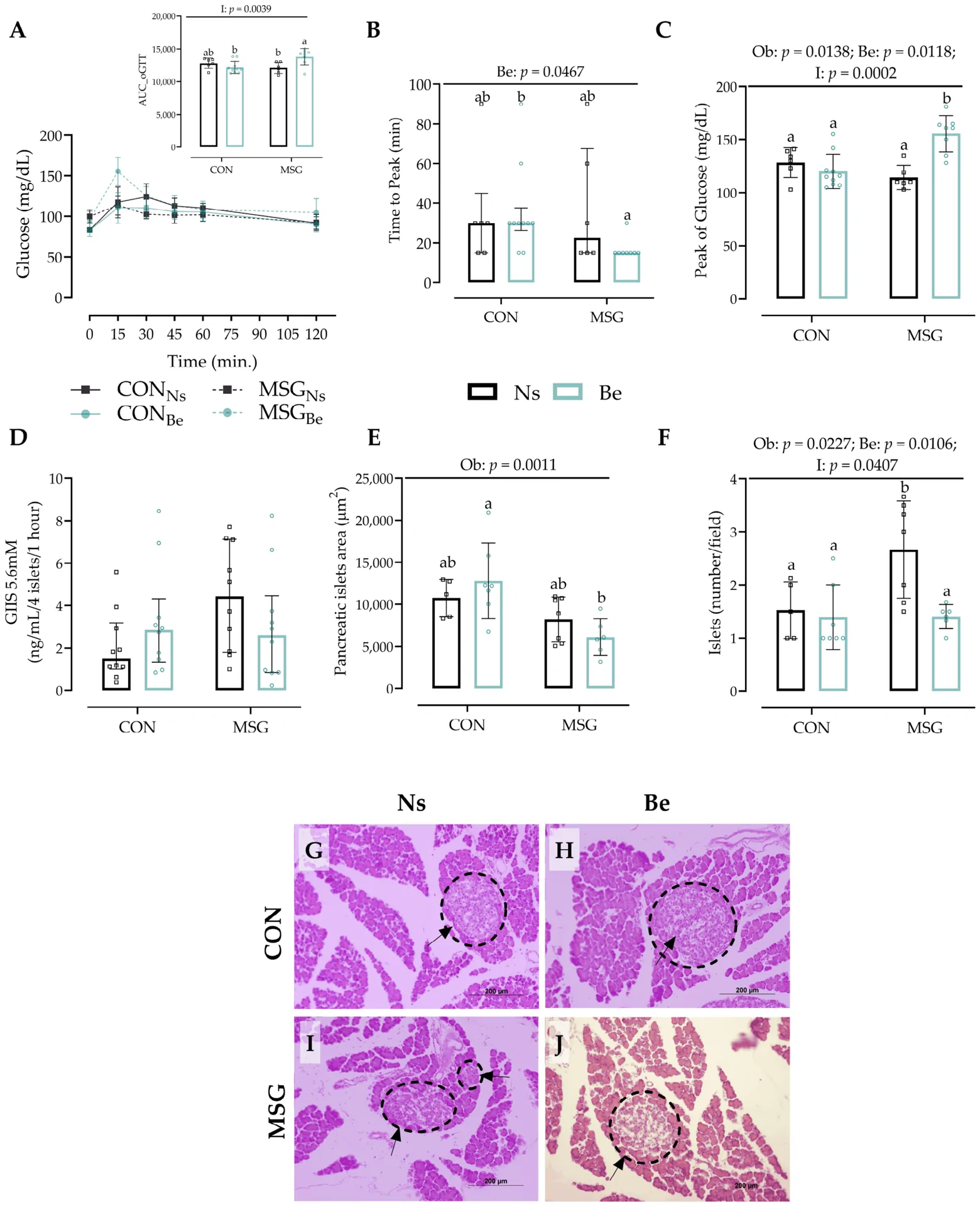
Effects of oral Be-oil supplementation on glucose homeostasis and islet histology in CON (lean) and MSG-obese male Wistar rats. (**A**) Glycemia during oGTT times and respective AUC (insert); (**B**) time to peak of oGTT (min); (**C**) peak of glucose (mg/dL); (**D**) GIIS (5.6 mM); (**E**) pancreatic islet area (µm^2^); (**F**) pancreatic islets (number/field). (**G**–**J**) Pancreatic photomicrographs (H&E-stained; scale bar = 200 μm; 20×). Black arrows indicate pancreatic islets, and dotted circles delineate the islet area. In Figure (**A**,**C**,**E**,**F**) data are expressed as mean ± SD (*n* = 5–10 rats per group). Two-way ANOVA with *p*-values of the factor (**F**) are indicated on the lines above the graphs: Ob: obesity; Be: Be-oil supplementation; I: interaction (Ob vs. Be). Different letters above the bars indicate statistical differences between the groups in the post hoc Tukey test (*p* < 0.05). Groups that share at least one letter are not significantly different, while groups with different letters are significantly different. In Figure (**B**,**D**), data are expressed as median with IQR. GLM.

**Figure 7:**
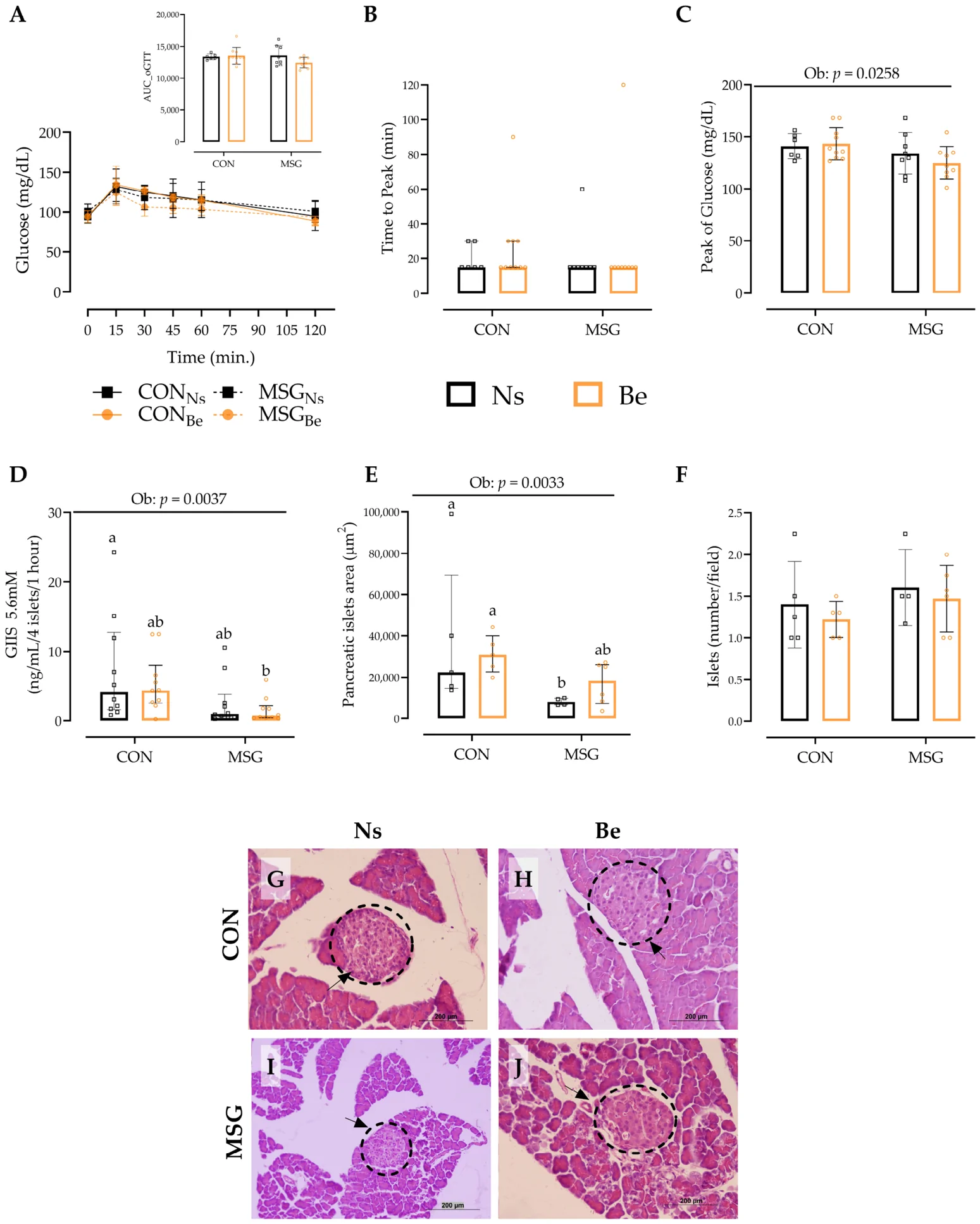
Effects of oral Be-oil supplementation on glucose homeostasis and islet histology in CON (lean) and MSG-obese female Wistar rats. (**A**) Glycemia during oGTT times and respective AUC (insert); (**B**) time to peak of oGTT (min); (**C**) peak of glucose (mg/dL); (**D**) GIIS (5.6 mM); (**E**) pancreatic islet area (µm^2^); (**F**) pancreatic islets (number/field). (**G**–**J**) Pancreatic photomicrographs (H&E-stained; scale bar = 200 μm; 20×). Black arrows indicate pancreatic islets, and dotted circles delineate the islet area. In Figure (**A**,**C**,**F**), data are expressed as mean ± SD (*n* = 5–10 rats per group). Two-way ANOVA with *p*-values of the factor (**F**) are indicated on the lines above the graphs: Ob: obesity; Be: Be-oil supplementation; I: interaction (Ob vs. Be). Different letters above the bars indicate statistical differences between the groups in the post hoc Tukey test (*p* < 0.05). Groups that share at least one letter are not significantly different, while groups with different letters are significantly different. In Figure (**B**,**D**,**E**), data are expressed as median with IQR. GLM.

## Data Availability

The original contributions presented in this study are included in the article. Further inquiries can be directed to the corresponding author.

## Supplementary Materials

The following supporting information can be downloaded at: https://www.mdpi.com/article/10.3390/biomedicines14102284/s1, Table S1. Physicochemical characteristics and fatty acid composition of Brazil nut oil (*Bertholletia excelsa*). Table S2. Effects of oral Be-oil supplementation on HOMA-β in male and female lean and MSG-obese Wistar rats at PND90.

## Abbreviations

The following abbreviations are used in this manuscript:

ALFAC	Alcohol–Formaldehyde–Acetic Acid Fixative
ARC	Arcuate Nucleus
AUC	Area Under the Curve
BAT	Brown Adipose Tissue
BMI	Body Mass Index
BW	Body Weight
CEUA	Animal Ethics Committee (Comitê de Ética no Uso de Animais)
CON	Control group
CONCEA	Brazilian National Council for the Control of Animal Experiments
FER	Feed Efficiency Ratio
FI	Food Intake
GIIS	Glucose-Induced Insulin Secretion
GLM	Generalized Linear Model
H&E	Hematoxylin and Eosin
HBS	Hank’s Buffer Solution
HDL-c	High-Density Lipoprotein Cholesterol
HOMA-β	Homeostasis Model Assessment of β-cell Function
HOMA-IR	Homeostasis Model Assessment of Insulin Resistance
iBAT	Interscapular Brown Adipose Tissue
IL6	Interleukin 6
IQR	Interquartile Range
IR	Insulin Resistance
KRB	Krebs–Ringer Buffer
LDL-c	Low-Density Lipoprotein Cholesterol
Ln	Natural Logarithm
MS	Metabolic Syndrome
MSG	Monosodium Glutamate
MUFA	Monounsaturated Fatty Acid
Ns	Non-supplemented
oGTT	Oral Glucose Tolerance Test
PND	Postnatal Day
PUFA	Polyunsaturated Fatty Acid
RIA	Radioimmunoassay
Scly	Selenocysteine Lyase
SD	Standard Deviation
T2D	Type 2 Diabetes
TNF-α	Tumor Necrosis Factor-α
TyG	Triglyceride–Glucose Index
WAT	White Adipose Tissue
WAT-I	Inguinal White Adipose Tissue
WAT-P	Perigonadal White Adipose Tissue

## References

[1] Dhondge R.H., Agrawal S., Patil R., Kadu A., Kothari M. (2024). A Comprehensive Review of Metabolic Syndrome and Its Role in Cardiovascular Disease and Type 2 Diabetes Mellitus: Mechanisms, Risk Factors, and Management. Cureus.

[2] Madan J., Desai S., Moitra P., Salis S., Agashe S., Battalwar R., Mehta A., Kamble R., Kalita S., Phatak A.G. (2021). Effect of Almond Consumption on Metabolic Risk Factors—Glucose Metabolism, Hyperinsulinemia, Selected Markers of Inflammation: A Randomized Controlled Trial in Adolescents and Young Adults. Front. Nutr..

[3] Estivaleti J.M., Guzman-Habinger J., Lobos J., Azeredo C.M., Claro R., Ferrari G., Adami F., Rezende L.F.M. (2022). Time Trends and Projected Obesity Epidemic in Brazilian Adults between 2006 and 2030. Sci. Rep..

[4] Cominetti C., De Bortoli M.C., Purgatto E., Ong T.P., Moreno F.S., Garrido A.B., Cozzolino S.M.F. (2011). Associations between Glutathione Peroxidase-1 Pro198Leu Polymorphism, Selenium Status, and DNA Damage Levels in Obese Women after Consumption of Brazil Nuts. Nutrition.

[5] Bressan J. (2014). Cacahuete Alto-Oleico Aumenta la Termogénesis Inducida por la Dieta en. Nutr. Hosp..

[6] Godwin N., Roberts T., Hooshmand S., Kern M., Hong M.Y. (2019). Mixed Nuts May Promote Satiety While Maintaining Stable Blood Glucose and Insulin in Healthy, Obese, and Overweight Adults in a Two-Arm Randomized Controlled Trial. J. Med. Food.

[7] Yang J. (2009). Brazil Nuts and Associated Health Benefits: A Review. LWT Food Sci. Technol..

[8] Kim Y., Keogh J., Clifton P. (2017). Benefits of Nut Consumption on Insulin Resistance and Cardiovascular Risk Factors: Multiple Potential Mechanisms of Actions. Nutrients.

[9] De Araújo B.A.N., De Campos L.M.T.D.S., Souza G.T., Abreu G.D.M., De Almeida C.D.O.R.P., Figueiredo M.S. (2025). Brazil Nut (*Bertholletia excelsa* H.B.K.) and Brazil Nut Oil: Effects on Oxidative Stress and Potential Therapeutic Health Benefits from Human, Animal, and Cell Culture Models. Nutr. Rev..

[10] Costa E Silva L.M., Pereira De Melo M.L., Faro Reis F.V., Monteiro M.C., Dos Santos S.M., Quadros Gomes B.A., Meller Da Silva L.H. (2019). Comparison of the Effects of Brazil Nut Oil and Soybean Oil on the Cardiometabolic Parameters of Patients with Metabolic Syndrome: A Randomized Trial. Nutrients.

[11] Cardoso B.R., Duarte G.B.S., Reis B.Z., Cozzolino S.M.F. (2017). Brazil Nuts: Nutritional Composition, Health Benefits and Safety Aspects. Food Res. Int..

[12] de Oliveira B.R., Egea M.B., Lemes S.A.d.F., Hernandes T., Takeuchi K.P. (2024). Beverage of Brazil Nut and Bocaiuva Almond Enriched with Minerals: Technological Quality and Nutritional Effect in Male Wistar Rats. Foods.

[13] Kume W.T., Jesus Porto E.P., Lara Spada E.C., Lisboa D.R., Stachack F.F.F., Terezo A.J., Hernandes T., Takeuchi K.P., Santos Elias M.P., Gai B.M. (2021). Acute Supplementation of Growing Rats with Brazil Nut Flour Increases Hepatic Lipid Content but Prevents Oxidative Damage in the Liver. J. Food Biochem..

[14] Da Silva Costa N., Almeida P.P., Da Cruz B.O., Brito M.L., Maldonado-Campos J., Menezes A.C., Figueiredo M.S., Magliano D.A.C., Pereira A.D., Stockler-Pinto M.B. (2022). Supplementation of Diet with Brazil Nut Modulates Body Composition, Bone Parameters, and Lipid Peroxidation in Wistar Rats. J. Food Biochem..

[15] Saldanha Melo H., Monnerat J.A.D.S., Costa N.D.S., Bento Bernardes T., Magliano D.C., Pereira A.D., Almeida P.P., Lima G.F., Ferreira De Brito F.C., Stockler Pinto M.B. (2022). Impact of Brazil Nut (*Bertholletia excelsa*, H.B.K.) Supplementation on Body Composition, Blood Pressure, and the Vascular Reactivity of Wistar Rats When Submitted to a Hypersodium Diet. J. Am. Nutr. Assoc..

[16] Nascimento L.P.S., Pires V.C., Ribeiro D.A., Gollücke A.P.B., Yamamura H., Junior O.A. (2020). Benefits of the Consumption of Brazil Nut (*Bertholletia excelsa*) Extract in Male Reproductive Parameters of Streptozotocin-Induced Diabetic Rats. J. Diabetes Metab. Disord..

[17] Menezes A.C., Brito M.L., Almeida P.P., Da Cruz B.O., Da Silva Costa N., D’Avila Pereira A., Castañon C., Nunes Degani V.A., Medeiros De França Cardozo L.F., Magliano D.C. (2024). Brazil Nuts Potential: Effects on Lipid Peroxidation and Heart Health in Nephrectomized Rats. J. Complement. Integr. Med..

[18] Costa M.A.D.C., Hermsdorff H.H.M., Caldas A.P.S., Rocha D.M.U.P., Da Silva A., De Oliveira L.L., Bressan J. (2021). Acute Consumption of a Shake Containing Cashew and Brazil Nuts Did Not Affect Appetite in Overweight Subjects: A Randomized, Cross-over Study. Eur. J. Nutr..

[19] Vilela D.L.D.S., Silva A.D., Pelissari Kravchychyn A.C., Bressan J., Hermsdorff H.H.M. (2024). Effect of Nuts Combined with Energy Restriction on the Obesity Treatment: A Systematic Review and Meta-Analysis of Randomized Controlled Trials. Foods.

[20] Fernández-Rodríguez R., Mesas A.E., Garrido-Miguel M., Martínez-Ortega I.A., Jiménez-López E., Martínez-Vizcaíno V. (2021). The Relationship of Tree Nuts and Peanuts with Adiposity Parameters: A Systematic Review and Network Meta-Analysis. Nutrients.

[21] Duarte G.B.S., Reis B.Z., Rogero M.M., Vargas-Mendez E., Júnior F.B., Cercato C., Cozzolino S.M.F. (2019). Consumption of Brazil Nuts with High Selenium Levels Increased Inflammation Biomarkers in Obese Women: A Randomized Controlled Trial. Nutrition.

[22] Torrezan R., Malta A., De Souza Rodrigues W.D.N., Dos Santos A.A.A., Miranda R.A., Moura E.G., Lisboa P.C., De Freitas Mathias P.C. (2019). Monosodium l-glutamate-obesity Onset Is Associated with Disruption of Central Control of the Hypothalamic-pituitary-adrenal Axis and Autonomic Nervous System. J. Neuroendocrinol..

[23] Villagarcía H.G., Castro M.C., Arbelaez L.G., Schinella G., Massa M.L., Spinedi E., Francini F. (2018). N-Acetyl-l-Cysteine Treatment Efficiently Prevented Pre-Diabetes and Inflamed-Dysmetabolic Liver Development in Hypothalamic Obese Rats. Life Sci..

[24] Svidnicki P.V., De Carvalho Leite N., Venturelli A.C., Camargo R.L., Vicari M.R., De Almeida M.C., Artoni R.F., Nogaroto V., Grassiolli S. (2013). Swim Training Restores Glucagon-like Peptide-1 Insulinotropic Action in Pancreatic Islets from Monosodium Glutamate-Obese Rats. Acta Physiol..

[25] Guareschi Z.M., Valcanaia A.C., Ceglarek V.M., Hotz P., Amaral B.K., De Souza D.W., De Souza T.A., Nardelli T., Ferreira T.R., Leite N.C. (2019). The Effect of Chronic Oral Vitamin D Supplementation on Adiposity and Insulin Secretion in Hypothalamic Obese Rats. Br. J. Nutr..

[26] Millard W.J., Martin J.B., Audet J., Sagar S.M., Martin J.B. (1982). Evidence That Reduced Growth Hormone Secretion Observed in Monosodium Glutamate-Treated Rats Is the Result of a Deficiency in Growth Hormone-Releasing Factor. Endocrinology.

[27] Roth C.L., McCormack S.E. (2024). Acquired Hypothalamic Obesity: A Clinical Overview and Update. Diabetes Obes. Metab..

[28] Donini L.M., Busetto L., Bischoff S.C., Cederholm T., Ballesteros-Pomar M.D., Batsis J.A., Bauer J.M., Boirie Y., Cruz-Jentoft A.J., Dicker D. (2022). Definition and Diagnostic Criteria for Sarcopenic Obesity: ESPEN and EASO Consensus Statement. Obes. Facts.

[29] Pizzi W.J., Barnhart J.E., Fanslow D.J. (1977). Monosodium Glutamate Administration to the Newborn Reduces Reproductive Ability in Female and Male Mice. Science.

[30] Anderson W.D., Soh J.Y., Innis S.E., Dimanche A., Ma L., Langefeld C.D., Comeau M.E., Das S.K., Schadt E.E., Björkegren J.L.M. (2020). Sex Differences in Human Adipose Tissue Gene Expression and Genetic Regulation Involve Adipogenesis. Genome Res..

[31] Rudyk M.P., Pozur V.V., Voieikova D.O., Hurmach Y.V., Khranovska N.M., Skachkova O.V., Svyatetska V.M., Fedorchuk O.G., Skivka L.M., Berehova T.V. (2018). Sex-Based Differences in Phagocyte Metabolic Profile in Rats with Monosodium Glutamate-Induced Obesity. Sci. Rep..

[32] Goossens G.H., Jocken J.W.E., Blaak E.E. (2021). Sexual Dimorphism in Cardiometabolic Health: The Role of Adipose Tissue, Muscle and Liver. Nat. Rev. Endocrinol..

[33] McArthur B., Considine R., Mattes R. (2018). Mastication of Nuts under Realistic Eating Conditions: Implications for Energy Balance. Nutrients.

[34] Colpo E., Vilanova C.D.D.A., Brenner Reetz L.G., Medeiros Frescura Duarte M.M., Farias I.L.G., Irineu Muller E., Muller A.L.H., Moraes Flores E.M., Wagner R., Da Rocha J.B.T. (2013). A Single Consumption of High Amounts of the Brazil Nuts Improves Lipid Profile of Healthy Volunteers. J. Nutr. Metab..

[35] Percie Du Sert N., Hurst V., Ahluwalia A., Alam S., Avey M.T., Baker M., Browne W.J., Clark A., Cuthill I.C., Dirnagl U. (2020). The ARRIVE Guidelines 2.0: Updated Guidelines for Reporting Animal Research. PLoS Biol..

[36] Olney J.W. (1969). Brain Lesions, Obesity, and Other Disturbances in Mice Treated with Monosodium Glutamate. Science.

[37] Quitian-Useche Y.F., Sánchez-Ortiz B.L., Borges S.F., Ramos B., De Souza G.C., Batista M.A., Da Silva Hage Melim L.I., Ferreira I.M., Carvalho J.C.T., Borges R.S. (2021). Fatty Ethanolamide of *Bertholletia excelsa* Triglycerides (Brazil Nuts): Anti-Inflammatory Action and Acute Toxicity Evaluation in Zebrafish (*Danio rerio*). Inflammopharmacology.

[38] Falcone A.P.D.M., Dutra L.M.G., Alves M.E.F., De Lima E Silva T.D.O., De Araújo J.M.D., Silva D.L.S., Martins G.V.F., Freita J.C.R., Viera V.B., Soares J.K.B. (2026). Supplementation with Refined Brazil Nut Oil (*Bertholletia excelsa*) during Pregnancy and Lactation Improves Physical, Biochemical and Hepatic Parameters in Rat Offspring. J. Nutr. Biochem..

[39] Nair A., Jacob S. (2016). A Simple Practice Guide for Dose Conversion between Animals and Human. J. Basic Clin. Pharm..

[40] Ni Z., Yang L., Qu M., Li Z., Tang F. (2023). Elemental Characterisation and Migration of Six Tree Nut Oils from Seed to Oil Under Physical Pressing.

[41] Carrillo J.-C., Shen H., Momin F., Kral O., Schnieder H., Kühn S. (2022). GTL Synthetic Paraffin Oil Shows Low Liver and Tissue Retention Compared to Mineral Oil. Food Chem. Toxicol..

[42] Chapman D.G., Castillo R., Campbell J.A. (1959). Evaluation of Protein in Foods: 1. A Method for The Determination of Protein Efficiency Ratios. Can. J. Biochem. Physiol..

[43] Kramer C.K., Vuksan V., Choi H., Zinman B., Retnakaran R. (2014). Emerging Parameters of the Insulin and Glucose Response on the Oral Glucose Tolerance Test: Reproducibility and Implications for Glucose Homeostasis in Individuals with and without Diabetes. Diabetes Res. Clin. Pract..

[44] Siqueira B.S., Díaz Urrutia M.A., Ceglarek V.M., Moreira D.C., Brasil Kuzniewski F.T., De Almeida Leite J.R.D.S., Grassiolli S. (2025). A Novel Bombesin-Related Peptide Modulates Glucose Tolerance and Insulin Secretion in Non-Obese and Hypothalamic-Obese Rats. Toxicon.

[45] Bernardis L.L., Patterson B.D. (1968). Correlation between “Lee Index” and Carcass Fat Content in Weanling and Adult Female Rats with Hypothalamic Lesions. J. Endocrinol..

[46] Lacy P.E., Kostianovsky M. (1967). Method for the Isolation of Intact Islets of Langerhans from the Rat Pancreas. Diabetes.

[47] Grassiolli S., Gravena C., De Freitas Mathias P.C. (2007). Muscarinic M2 Receptor Is Active on Pancreatic Islets from Hypothalamic Obese Rat. Eur. J. Pharmacol..

[48] Yalow R.S., Berson S.A. (1960). Immunoassay of Endogenous Plasma Insulin in Man. J. Clin. Investig..

[49] Simental-Mendía L.E., Rodríguez-Morán M., Guerrero-Romero F. (2008). The Product of Fasting Glucose and Triglycerides as Surrogate for Identifying Insulin Resistance in Apparently Healthy Subjects. Metab. Syndr. Relat. Disord..

[50] Matthews D.R., Hosker J.P., Rudenski S., Treacher D.F. (1985). Homeostasis Model Assessment: Insulin Resistance and Beta-Cell Function from Fasting Plasma Glucose and Insulin Concentrations in Man. Diabetologia.

[51] Guareschi Z.M.N.D.C., Urrutia M.D., Daudt B.M., Siqueira B.S., Ceglarek V.M., Dos Santos M.E., Grassiolli S. (2025). The Metabolic and Anti-Adiposity Effects of Oral Copaiba-Oil Supplementation Are Influenced by Sex and Obesity. Nutrivisa.

[52] De Souza D.W., Ceglarek V.M., Siqueira B.S., Volinski C.Z., Nenevê J.Z., Arruda J.P.D.A., Vettorazzi J.F., Grassiolli S. (2022). Phenylhydrazine-induced Anaemia Reduces Subcutaneous White and Brown Adipose Tissues in Hypothalamic Obese Rats. Exp. Physiol..

[53] Rodrigues W.N.D.S., Malta A., Lucredi N.C., Lisboa P.C., Miranda R.A., Vargas R., Zara C.B., Peres M.N.C., Teixeira Junior N.R., Comar J.F. (2025). Adrenalectomy Improves MSG-Induced Obesity in Rats by Increasing UCP-1 Levels in Interscapular Brown Adipose Tissue. Acta Sci. Biol. Sci..

[54] Gaspar R.S., Benevides R.O.A., de Lima Fontelles J.L., Vale C.C., França L.M., de Tarso Silva Barros P., de Andrade Paes A.M. (2016). Reproductive Alterations in Hyperinsulinemic but Normoandrogenic MSG Obese Female Rats. J. Endocrinol..

[55] Siqueira B.S., Gomes E.C.Z., Rentz T., Malta A., De Freitas Mathias P.C., Balbo S.L., Grassiolli S. (2025). Vagal Splenic-Dependent Effects Influence Glucose Homeostasis, Insulin Secretion, and Histopathology of the Endocrine Pancreas in Hypothalamic Obese Male Rats: Vagus Nerve and Spleen Interactions Affect the Endocrine Pancreas. Sci. World J..

[56] Zazula M.F., Saraiva D.F., Theodoro J.L., Maciel M., Sepulveda E.V.D.S., Zanardini de Andrade B., Boaretto M.L., Maciel J.I.H.N., Bronczek G.A., Soares G.M. (2023). An Early and Sustained Inflammatory State Induces Muscle Changes and Establishes Obesogenic Characteristics in Wistar Rats Exposed to the MSG-Induced Obesity Model. Int. J. Mol. Sci..

[57] Andreazzi A.E., Scomparin D.X., Mesquita F.P., Balbo S.L., Gravena C., Oliveira J.C.D., Rinaldi W., Garcia R.M.G., Grassiolli S., Mathias P.C.F. (2009). Swimming Exercise at Weaning Improves Glycemic Control and Inhibits the Onset of Monosodium L-Glutamate-Obesity in Mice. J. Endocrinol..

[58] Ribeiro R.A., Bonfleur M.L., Vanzela E.C., Zotti A.I., Scomparin D.X., Boschero A.C., Balbo S.L. (2014). Physical Exercise Introduced After Weaning Enhances Pancreatic Islet Responsiveness to Glucose and Potentiating Agents in Adult MSG-Obese Rats. Horm. Metab. Res..

[59] Araujo T.R., Freitas I.N., Vettorazzi J.F., Batista T.M., Santos-Silva J.C., Bonfleur M.L., Balbo S.L., Boschero A.C., Carneiro E.M., Ribeiro R.A. (2017). Benefits of L-Alanine or l-Arginine Supplementation against Adiposity and Glucose Intolerance in Monosodium Glutamate-Induced Obesity. Eur. J. Nutr..

[60] Leite N.D.C., Montes E.G., Fisher S.V., Cancian C.R.C., De Oliveira J.C., Martins-Pinge M.C., Kanunfre C.C., Souza K.L.A., Grassiolli S. (2015). Splenectomy Attenuates Obesity and Decreases Insulin Hypersecretion in Hypothalamic Obese Rats. Metabolism.

[61] Boušová I., Matoušková P., Bártíková H., Szotáková B., Hanušová V., Tománková V., Anzenbacherová E., Lišková B., Anzenbacher P., Skálová L. (2016). Influence of Diet Supplementation with Green Tea Extract on Drug-Metabolizing Enzymes in a Mouse Model of Monosodium Glutamate-Induced Obesity. Eur. J. Nutr..

[62] Maranhão P.A., Kraemer-Aguiar L.G., De Oliveira C.L., Kuschnir M.C., Vieira Y.R., Souza M.G., Koury J.C., Bouskela E. (2011). Brazil Nuts Intake Improves Lipid Profile, Oxidative Stress and Microvascular Function in Obese Adolescents: A Randomized Controlled Trial. Nutr. Metab..

[63] Macan T.P., De Amorim T.A., Damiani A.P., Beretta Â.C.D.L., Magenis M.L., Vilela T.C., Teixeira J.P., Andrade V.M.D. (2022). Brazil Nut Prevents Oxidative DNA Damage in Type 2 Diabetes Patients. Drug Chem. Toxicol..

[64] Da Silva A., Silveira B.K.S., De Freitas B.V.M., Hermsdorff H.H.M., Bressan J. (2022). Effects of Regular Brazil Nut (*Bertholletia excelsa* H.B.K.) Consumption on Health: A Systematic Review of Clinical Trials. Foods.

[65] Pena Muniz M., Ferreira Dos Santos M., Da Costa C.E., Morais L., Lamarão M.N., Ribeiro-Costa R., Silva J.O. (2015). Physicochemical Characterization, Fatty Acid Composition, and Thermal Analysis of *Bertholletia excelsa* HBK Oil. Pharmacogn. Mag..

[66] Benoit S.C., Kemp C.J., Elias C.F., Abplanalp W., Herman J.P., Migrenne S., Lefevre A.-L., Cruciani-Guglielmacci C., Magnan C., Yu F. (2009). Palmitic Acid Mediates Hypothalamic Insulin Resistance by Altering PKC-θ Subcellular Localization in Rodents. J. Clin. Investig..

[67] Frausto-González O., Bautista C.J., Narváez-González F., Hernandez-Leon A., Estrada-Camarena E., Rivero-Cruz F., González-Trujano M.E. (2021). *Bertholletia excelsa* Seeds Reduce Anxiety-Like Behavior, Lipids, and Overweight in Mice. Molecules.

[68] Rudyk M., Hurmach Y., Serhiichuk T., Akulenko I., Skivka L., Berehova T., Ostapchenko L. (2023). Multi-Probiotic Consumption Sex-Dependently Interferes with MSG-Induced Obesity and Concomitant Phagocyte pro-Inflammatory Polarization in Rats: Food for Thought about Personalized Nutrition. Heliyon.

[69] Cavaliere G., Trinchese G., Bergamo P., De Filippo C., Mattace Raso G., Gifuni G., Putti R., Moni B.H., Canani R.B., Meli R. (2016). Polyunsaturated Fatty Acids Attenuate Diet Induced Obesity and Insulin Resistance, Modulating Mitochondrial Respiratory Uncoupling in Rat Skeletal Muscle. PLoS ONE.

[70] Huguenin G.V.B., Oliveira G.M.M., Moreira A.S.B., Saint’Pierre T.D., Gonçalves R.A., Pinheiro-Mulder A.R., Teodoro A.J., Luiz R.R., Rosa G. (2015). Improvement of Antioxidant Status after Brazil Nut Intake in Hypertensive and Dyslipidemic Subjects. Nutr. J..

[71] La Grasta Sabolić L., Šepec M.P., Cigrovski Berković M., Stipančić G. (2021). Time to the Peak, Shape of the Curve and Combination of These Glucose Response Characteristics During Oral Glucose Tolerance Test as Indicators of Early Beta-Cell Dysfunction in Obese Adolescents. J. Clin. Res. Pediatr. Endocrinol..

[72] Chung S.T., Ha J., Onuzuruike A.U., Kasturi K., Galvan-De La Cruz M., Bingham B.A., Baker R.L., Utumatwishima J.N., Mabundo L.S., Ricks M. (2017). Time to Glucose Peak during an Oral Glucose Tolerance Test Identifies Prediabetes Risk. Clin. Endocrinol..

[73] Cardoso B.R., Braat S., Graham R.M. (2021). Selenium Status Is Associated with Insulin Resistance Markers in Adults: Findings from the 2013 to 2018 National Health and Nutrition Examination Survey (NHANES). Front. Nutr..

[74] Furse S., Virtue S., Huang-Doran I., Vidal-Puig A., Chiarugi D., Stevenson P.C., Snowden S.G., Koulman A. (2025). Systemic Analyses Show That the Biosynthesis and Spatial Distribution of Fatty Acids, Triglycerides and Lipids Differed in Male and Female Mice and Humans. Open Biol..

[75] Gao A., Su J., Liu R., Zhao S., Li W., Xu X., Li D., Shi J., Gu B., Zhang J. (2021). Sexual Dimorphism in Glucose Metabolism Is Shaped by Androgen-Driven Gut Microbiome. Nat. Commun..

[76] Rodrigues R.C.B., Nascimento D., Brito J., Lima L.S., Lobo J.C., Cardozo L.F.M.F., Mafra D. (2025). Brazil Nut or Not? Uncovering the Best Source of Selenium for Chronic Non-Communicable Disease. Food Biosci..

[77] Seale L.A., Hashimoto A.C., Kurokawa S., Gilman C.L., Seyedali A., Bellinger F.P., Raman A.V., Berry M.J. (2012). Disruption of the Selenocysteine Lyase-Mediated Selenium Recycling Pathway Leads to Metabolic Syndrome in Mice. Mol. Cell. Biol..

[78] Jablonska E., Reszka E., Gromadzinska J., Wieczorek E., Krol M.B., Raimondi S., Socha K., Borawska M.H., Wasowicz W. (2016). The Effect of Selenium Supplementation on Glucose Homeostasis and the Expression of Genes Related to Glucose Metabolism. Nutrients.

[79] Donadio J.L.S., Rogero M.M., Guerra-Shinohara E.M., Desmarchelier C., Borel P., Cozzolino S.M.F. (2018). SEPP1 Polymorphisms Modulate Serum Glucose and Lipid Response to Brazil Nut Supplementation. Eur. J. Nutr..

[80] Ogawa-Wong A., Hashimoto A., Ha H., Pitts M., Seale L., Berry M. (2018). Sexual Dimorphism in the Selenocysteine Lyase Knockout Mouse. Nutrients.

[81] Cominetti C., de Bortoli M.C., Garrido A.B., Cozzolino S.M.F. (2012). Brazilian Nut Consumption Improves Selenium Status and Glutathione Peroxidase Activity and Reduces Atherogenic Risk in Obese Women. Nutr. Res..

[82] Godos J., Giampieri F., Micek A., Battino M., Forbes-Hernández T.Y., Quiles J.L., Paladino N., Falzone L., Grosso G. (2022). Effect of Brazil Nuts on Selenium Status, Blood Lipids, and Biomarkers of Oxidative Stress and Inflammation: A Systematic Review and Meta-Analysis of Randomized Clinical Trials. Antioxidants.

[83] Vivoli A., Ghislain J., Filali-Mouhim A., Angeles Z.E., Castell A.-L., Sladek R., Poitout V. (2022). Single-Cell RNA Sequencing Reveals a Role for Reactive Oxygen Species and Peroxiredoxins in Fatty Acid–Induced Rat β-Cell Proliferation. Diabetes.

[84] Manukyan L., Ubhayasekera S.J.K.A., Bergquist J., Sargsyan E., Bergsten P. (2015). Palmitate-Induced Impairments of β-Cell Function Are Linked with Generation of Specific Ceramide Species via Acylation of Sphingosine. Endocrinology.

[85] Nemecz M., Constantin A., Dumitrescu M., Alexandru N., Filippi A., Tanko G., Georgescu A. (2018). The Distinct Effects of Palmitic and Oleic Acid on Pancreatic Beta Cell Function: The Elucidation of Associated Mechanisms and Effector Molecules. Front. Pharmacol..

[86] Chunhieng T., Hafidi A., Pioch D., Brochier J., Didier M. (2008). Detailed Study of Brazil Nut (*Bertholletia excelsa*) Oil Micro-Compounds: Phospholipids, Tocopherols and Sterols. J. Braz. Chem. Soc..

[87] Cicero N., Albergamo A., Salvo A., Bua G.D., Bartolomeo G., Mangano V., Rotondo A., Di Stefano V., Di Bella G., Dugo G. (2018). Chemical Characterization of a Variety of Cold-Pressed Gourmet Oils Available on the Brazilian Market. Food Res. Int..

[88] Guo L., Nan Y., Liang K., Yao L., Li J. (2024). Association between Polyunsaturated Fatty Acid Intake and Estradiol Levels among U.S. Women. Front. Nutr..

[89] Castellano C.-A., Audet I., Laforest J.-P., Matte J.J., Suh M. (2011). Fish Oil Diets Alter the Phospholipid Balance, Fatty Acid Composition, and Steroid Hormone Concentrations in Testes of Adult Pigs. Theriogenology.

[90] Maranesi M., Castellini C., Dall’Aglio C., Petrucci L., Mattioli S., Boiti C., Zerani M. (2018). Effects of PUFAs on Animal Reproduction: Male and Female Performances and Endocrine Mechanisms. Phytochem. Rev..

[91] Zucker I. (2023). The Mixed Legacy of the Rat Estrous Cycle. Biol. Sex Differ..

[92] Parker G.C., McKee M.E., Bishop C., Coscina D.V. (2001). Whole-Body Metabolism Varies across the Estrous Cycle in Sprague–Dawley Rats. Physiol. Behav..

[93] Kim S., Shin S., Chang Y., Sung E., Kang J.-H. (2026). Sex-Stratified Associations of TyG Index and HOMA-IR Changes with Incident Type 2 Diabetes. Sci. Rep..

