# Supplementary material for: Effect of the Long-Term Oral Consumption of Brazil Nut Oil on Adiposity and Glucose Homeostasis in MSG-Induced Obese Male and Female Rats
Source: Biomedicines. 2026 Oct 9;14(10):2284. doi: 10.3390/biomedicines14102284 (PMC13701298; doi:10.3390/biomedicines14102284)
Supplement: Supplementary file 1 [file biomedicines-14-02284-s001.zip › biomedicines-4480537-supplementary.pdf]

**Table S1.** Physicochemical characteristics and fatty acid composition of Brazil nut oil (*Bertholletia excelsa*).

| **Physicochemical Characteristics of Brazil Nut Oil** | | |
|---|---|---|
| **Characteristic** | Unit | Specification |
| Appearance (25 °C) | — | Liquid |
| Color | — | Translucent yellow |
| Odor | — | Characteristic |
| Acid value | mg KOH/g | < 20.0 |
| Peroxide value | meq $O_2$/kg | < 15.0 |
| Iodine value | g $I_2$/100 g | 90–110 |
| Saponification value | mg KOH/g | 180–210 |
| Density | g/mL at 25 °C | 0.910–0.998 |
| Refractive index (40 °C) | — | 1.4600–1.4800 |
| Unsaponifiable matter (bioactive compounds) | % | 0.5 |
| Melting point | °C | 4 |
| **Fatty Acid Composition** | | |
| Palmitic acid (C16:0) | % | 16.0–20.0 |
| Palmitoleic acid (C16:1) | % | 0.5–1.2 |
| Stearic acid (C18:0) | % | 9.0–13.0 |
| Oleic acid (C18:1, Omega-9) | % | 36.0–45.0 |
| Linoleic acid (C18:2, Omega-6) | % | 33.0–38.0 |
| Others | % | 4.0–6.0 |
| Saturated fatty acids | % | 25 |
| Unsaturated fatty acids | % | 75 |
| Se † 0.158μg/$Kg^{-1}$ | | |
| HED* 0.147mg/Kg | | |

The original table was provided in Portuguese by AmazonOil and was translated into English for presentation in this manuscript, according to the specifications available on the supplier's website. †Selenium(Se) concentration in Be-oil obtained by Zhanglin et al. [40]. These authors applied the same cold pressure extraction method to obtain Be-oil as that used by the AmazonOil company. *Human equivalent dose (HDE) according to Nair and Jacob [39]. At this dose toxicity effects would be unlikely.

**Table S2.** Effects of oral Be-oil supplementation on HOMA-β in male and female lean and MSG-obese Wistar rats at PND90.

| | **Groups** | | | | ***p*-Value (F)** | | |
|---|---|---|---|---|---|---|---|
| **Male** | **$CON_{Ns}$** | **$CON_{Be}$** | **$MSG_{Ns}$** | **$MSG_{Be}$** | **Ob** | **Be** | **I** |
| HOMA-β† | 90.4 [14.3] | 34.6[52.0] | 195[134] | 99.0[43.0] | <0.1445 (2.25) | 0.3722 (0.82) | 0.1891 (1.81) |
| **Female** | | | | | | | |
| HOMA-β† | 4.95[7.28]$^a$ | 13.7[48.5]$^{ab}$ | 18.1[18.2]$^a$ | 90.4 [138]$^b$ | <0.0270 (5.42) | 0.0190 (6.16) | 0.1164(2.62) |

† Data expressed as median with [IQR]. p-values of factor (F) effects obtained in the GLM are shown: Ob: obesity; Be: Be-oil supplementation; I: interaction (Ob × Be). p-value < 0.05. Different letters above the numbers indicate statistical differences between the groups on the post hoc Tukey test ($p < 0.05$). Groups that share at least one letter are not significantly different, while groups with different letters are significantly different. CON: control (lean); MSG: monosodium glutamate (obese); Ns: non-supplemented; Be: Be-oil-supplemented.
